# Wearable Biodevices Based on Two-Dimensional Materials: From Flexible Sensors to Smart Integrated Systems

**DOI:** 10.1007/s40820-024-01597-w

**Published:** 2025-01-15

**Authors:** Yingzhi Sun, Weiyi He, Can Jiang, Jing Li, Jianli Liu, Mingjie Liu

**Affiliations:** 1 School of Medical Technology, Beijing Institute of Technology, Beijing, 100081 People’s Republic of China; 2 Key Laboratory of Bio-Inspired Smart Interfacial Science and Technology of Ministry of Education, School of Chemistry, Beihang University, Beijing, 100191 People’s Republic of China

**Keywords:** Two-dimensional material, Wearable biodevice, Flexible sensor, Smart integrated system, Healthcare

## Abstract

The proliferation of wearable biodevices has boosted the development of soft, innovative, and multifunctional materials for human health monitoring. The integration of wearable sensors with intelligent systems is an overwhelming tendency, providing powerful tools for remote health monitoring and personal health management. Among many candidates, two-dimensional (2D) materials stand out due to several exotic mechanical, electrical, optical, and chemical properties that can be efficiently integrated into atomic-thin films. While previous reviews on 2D materials for biodevices primarily focus on conventional configurations and materials like graphene, the rapid development of new 2D materials with exotic properties has opened up novel applications, particularly in smart interaction and integrated functionalities. This review aims to consolidate recent progress, highlight the unique advantages of 2D materials, and guide future research by discussing existing challenges and opportunities in applying 2D materials for smart wearable biodevices. We begin with an in-depth analysis of the advantages, sensing mechanisms, and potential applications of 2D materials in wearable biodevice fabrication. Following this, we systematically discuss state-of-the-art biodevices based on 2D materials for monitoring various physiological signals within the human body. Special attention is given to showcasing the integration of multi-functionality in 2D smart devices, mainly including self-power supply, integrated diagnosis/treatment, and human–machine interaction. Finally, the review concludes with a concise summary of existing challenges and prospective solutions concerning the utilization of 2D materials for advanced biodevices.

## Highlights

- Two-dimensional (2D) materials are highlighted for their exceptional mechanical, electrical, optical, and chemical properties, making them ideal for fabricating high-performance wearable biodevices.
- The review categorizes cutting-edge wearable biodevices by their interactions with physical, electrophysiological, and biochemical signals, showcasing how 2D materials enhance these devices' functionality, mainly including self-powering and human-machine interaction.
- 2D materials enable multifunctional, high-performance biodevices, integrating self-powered systems, treatment platforms, and human-machine interactions, though challenges remain in practical applications.

## Graphical abstract

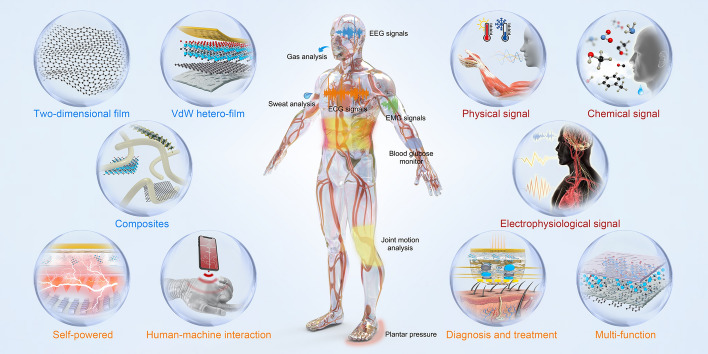

## Introduction

With the rising incidence of various diseases and the aging issue of the population worldwide, there is an unprecedented focus on health monitoring and medical care [1–3]. Wearable biodevices have emerged as a promising solution, offering feasible approaches to monitor a broad range of physical, electrophysiological, and biochemical signals regularly and continuously. These devices hold great potential for applications in preventive medicine, disease diagnosis, rehabilitation treatment, and daily health management [4–10]. Additionally, wearable biodevices not only enable the selective distinction of numerous health-related signals such as heart rate, body temperature, and blood glucose levels [11–15], but also allow for external stimulus or treatment via wireless integration with mobile electronics and internet big data [16–18].

In the early stages, significant efforts were directed towards utilizing inorganic (like silicon or metals) and organic materials for biodevice fabrication [19–21]. However, conventional hard electronic materials exhibit intrinsic mismatches with soft biological tissues in terms of electrical conductivity, mechanical response, permeability, and environmental adaptability [22]. While hard inorganic semiconductors can be rendered flexible in ultrathin membrane format, they are barely stretchable and cannot form a conformal interface with irregular geometries due to their fundamental topological limitations [23]. Deformation-tolerant structures, such as wrinkled [24], buckled [25], waved [26], or serpentine structures [27–29], address macroscopic stretchability but fail to provide microscopic conformability due to microscopic structural undulations. Organic or composite semiconductor thin films offer stretchability or conformability [30] but often suffer from insufficient electronic performance [23, 31] or limited stability in wet biological environments [32–35].

Ever since the landmark isolation of graphene monolayer in 2004 [36, 37], dozens of 2D monolayers have been extensively explored, including mono-element family (Xenes) [38–40], transition metal dichalcogenides (TMDs) [41–43], transition metal carbides and nitrides (MXenes) [44–46], layered double hydroxides (LDHs) [47, 48], and more [49]. The 2D monolayer, as the fundamental functional unit in layered bulk materials, largely retains its properties in atomic thickness, offering unparalleled advantages over other nanomaterials for applications in miniaturized devices [41, 50, 51]. Furthermore, the quantum confinement effect [52–54], diverse composition [46, 55], as well as facial assembly within the 2D material family [56, 57], further expand the possibilities for integrating versatile functionalities, including mechanics [58–60], conductivity [61–63], photonics [50, 64, 65], and so forth, into thin van der Waals heterostructures [66–68], enabling tailored design for multiple functional devices [69, 70].

**Fig. 1:**
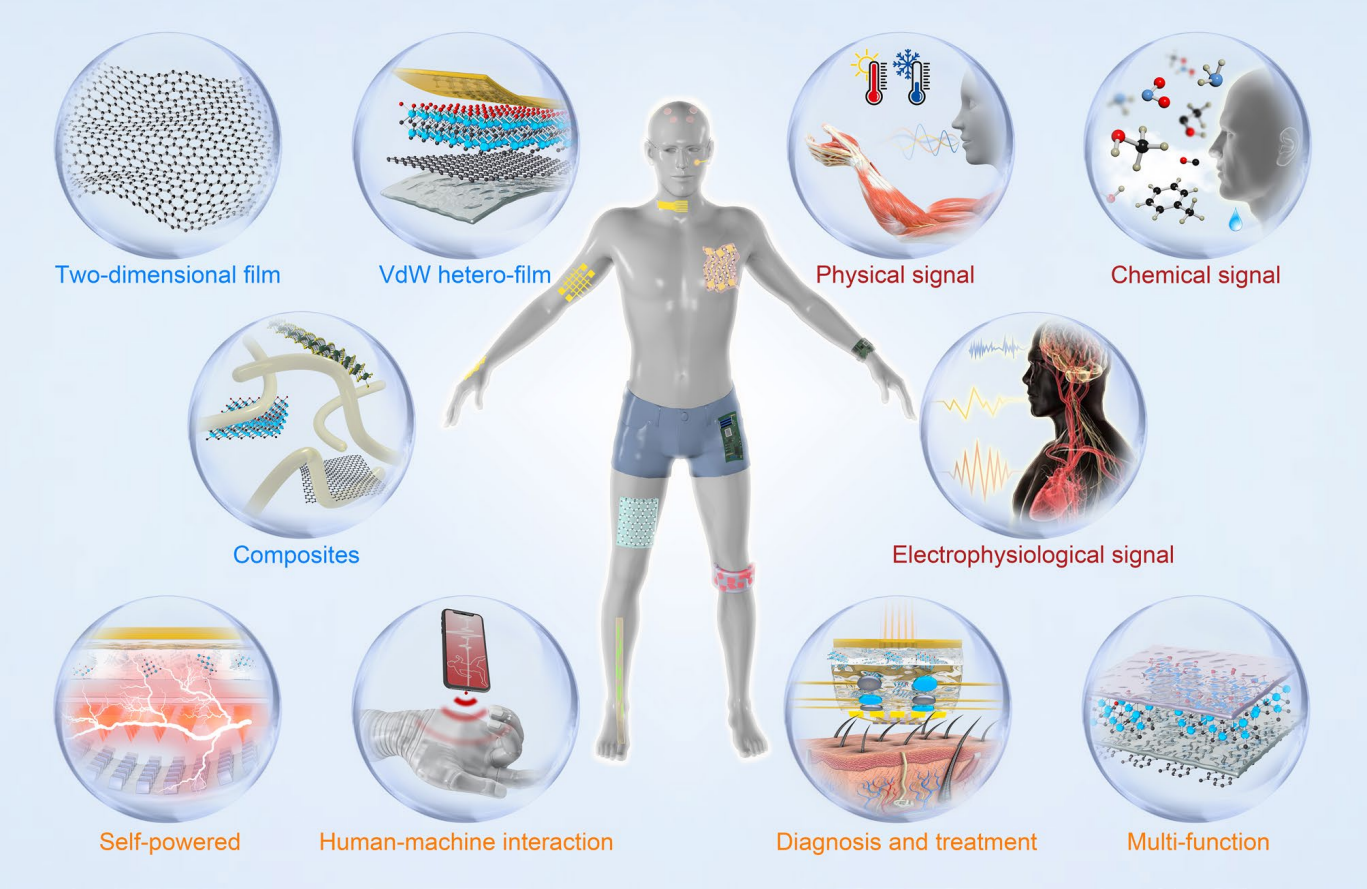
Overview of 2D material-based wearable devices and their health-related applications

Recently, the integration of 2D materials with wearable biodevices has attracted significant attention, resulting in remarkable advancements [71–73]. This review aims to comprehensively evaluate the current material and technological advances in 2D limits, as well as the future challenges for their potential applications. Firstly, the intrinsic properties of 2D materials were analyzed and their advantages in constructing wearable biodevices were outlined. Subsequently, the complex physiological signals of the human body were categorized into physical, electrophysiological, and chemical signals to showcase the latest applications of 2D materials in wearable biodevices. Then, intelligent wearable devices based on 2D materials were summarized, encompassing those integrated with self-powered systems, medical treatment platforms, and human–machine interactions. Finally, this review concludes with a brief discussion of the existing challenges and opportunities for applying 2D materials in smart wearable biodevices (Fig. 1).

## Advantages in 2D Materials for Wearable Biodevices

**Fig. 2:**
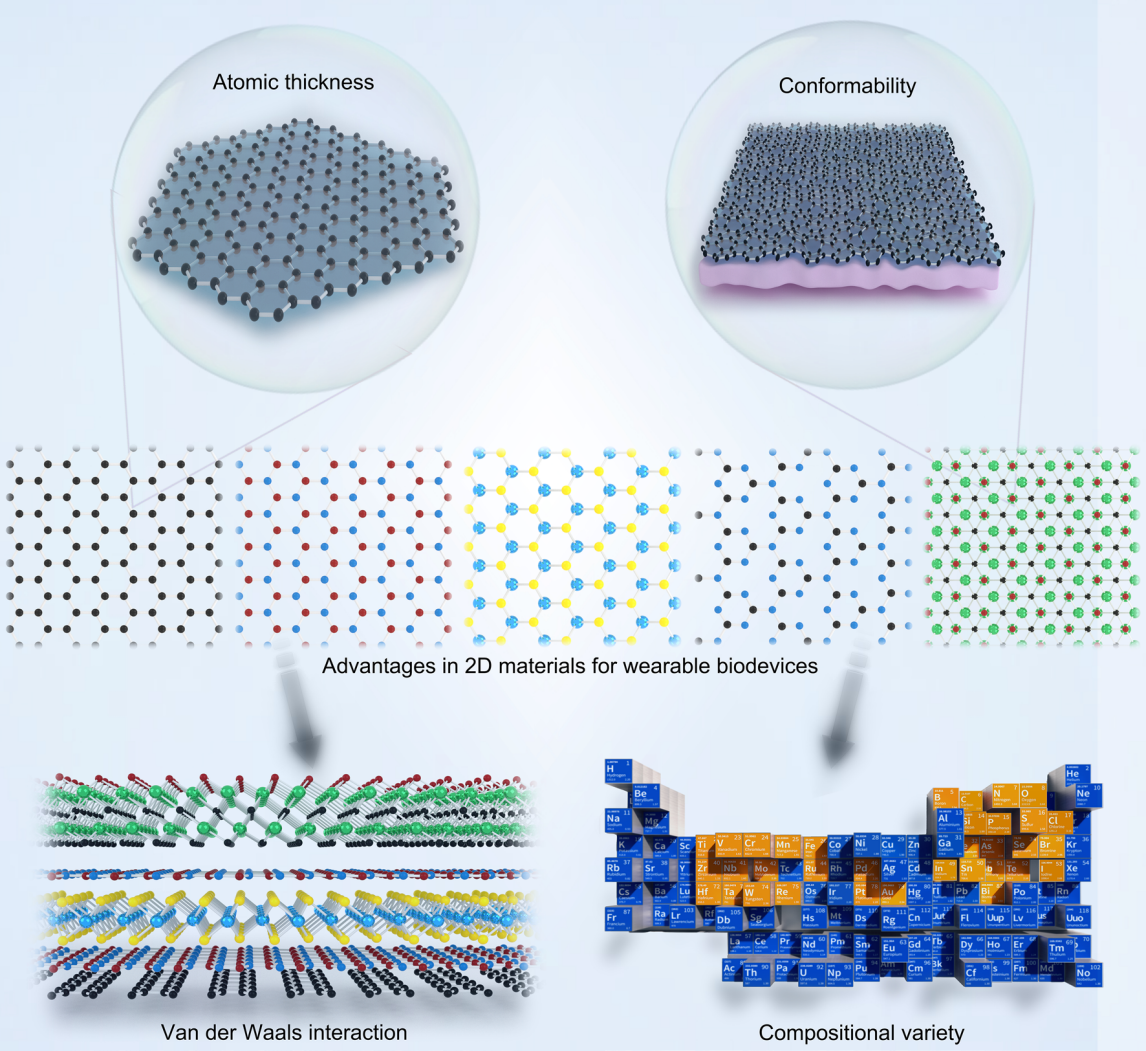
Advantages in 2D materials for wearable devices. 2D materials, with their atomic thickness, excel in these areas by reducing strain and offering superior flexibility and elasticity, allowing them to conform closely to human tissues. The van der Waals interactions in these materials also enable the creation of multifunctional heterostructures with customizable properties. Additionally, the vast range of 2D materials, incorporating various elements, provides extensive opportunities for innovation in wearable technology

Over the past decades, there has been a great enthusiasm in witnessing the advent of each new 2D material [49, 74], which has profoundly influenced a diverse landscape of bioelectronics [55, 75]. A prime exemplar is graphene, unearthed about twenty years ago, yet still exerting a pivotal influence on cutting-edge wearable devices such as transparent electrodes, flexible substrates, and a plethora of function-enhanced additives [76, 77]. This enduring significance primarily stems from its intrinsic *sp*^2^ bond configuration and a consequential array of exotic physical and chemical properties, like exceptional tensile strength (130 GPa) [58], remarkable thermal conductivity (5000 W m^−1^ K^−1^) [78], extraordinary carrier mobility (200,000 cm^2^ V^−1^ s^−1^) [79], outstanding optical transmittance (97.7%) [56], and huge theoretical specific surface area (2,630 m^2^ g^−1^) [58, 61]. The high surface-to-volume ratio and high flexibility of the 2D nanosheets endow the sensors with excellent portability and wearability [80]. The ongoing exploration of novel 2D materials in the realm of wearable biodevices [39], alongside the innovative utilization of traditional 2D materials in emerging biodevice applications [40], epitomizes two frontiers of contemporary research. Atomically thin 2D materials, characterized by their distinctive structures and intriguing properties, offer a competitive material platform for the fabrication of versatile biodevices [81, 82]. To elucidate the advantages and characteristics of wearable devices based on 2D materials, this section is initiated with an introduction of the structural aspects of 2D materials, followed by a comprehensive discussion of the diverse physical and chemical properties emanating from their structures, underscoring their newfound application in wearable biodevices (Fig. 2).

### Atomic Thickness

Deformability is a fundamental requisite in the construction of wearable devices. Given the intricate and continual movements of the human body, devising a robustly deformable material capable of withstanding diverse motion frequencies and amplitudes poses a significant challenge [3]. A commonly pursued strategy involves reducing material thickness, recognized as a generic approach to mitigate strain-induced structural cracks and bolster intrinsic deformability. For instance, in the context of the simplest one-dimensional bendable bio-devices employed on the wrist and fingers, the bending strain (*ε*) could be quantified as $\varepsilon = {\text{t}}/(2{\text{R}} + {\text{t}})$, where *t* denotes thickness and *R* signifies bending radius [83]. Reducing thickness from the micrometer scale typical of conventional metal or organic films to sub-nanometer dimensions achievable with 2D drastically alleviates strain concerns in biodevices by orders of magnitude. The reduction in dimensions and the increase in specific surface area of sensing materials can amplify their optical, electrical, and chemical properties. Therefore, low-dimensional nanomaterials with a high surface-to-volume ratio provide higher sensitivity and lower detection limits in sensing applications. For instance, atomic-layer-thick chemical vapor deposition (CVD) graphene shows strong σ bonds formed by in-plane sp^2^ hybridization, while the remaining p orbitals create a delocalized π electron structure, resulting in in-plane electrical conductivity that is several orders of magnitude higher than the conductivity in the vertical direction [84]. Consequently, the high conductivity of graphene enables outstanding performance in electronic devices, particularly in pressure sensors with rapid response times. For instance, Zhang et al. reported highly sensitive flexible pressure sensors based on CVD graphene that demonstrated high sensitivity ranging from 0 (kPa)^−2^ under pressures of 110–1.0 kPa [85]. Consequently, 2D materials emerge as generic building blocks for various wearable biodevices.

### Conformability

2D materials with atomic thickness and unique mechanical properties, enable sensors to maintain structural and performance stability under bending, stretching, or twisting, leading to good conformability to different surface shapes. For biodevices to function effectively in diverse environments and advance implantable applications, these devices must exhibit excellent conformability with target human tissues, enabling effective bio-signal monitoring and enduring application in diverse environments [3]. However, the inherent mechanical mismatch due to the inadequate compatibility or conformability of sensing materials presents a significant challenge for applying wearable tactile sensing devices in human–machine interaction [86]. To this end, the tunable stiffness and exceptional elasticity of nanomaterials are of paramount importance. The co-existence of atomic thickness and excellent mechanical properties in 2D materials [75] offers a vast family of competitive members for designing conformal biodevices. Firstly, a 2D monolayer stands out as the softest material, seamlessly conforming to various human tissues. The bending rigidity (*D*) in materials can be evaluated as ${\text{D}} = {\text{E}} {\text{t}}^{3} /12(1 {-} {\text{v}}^{2} )$, where *E* represents Young's modulus, *t* denotes thickness, and *v* signifies Poisson’s ratio [87]. The nanometer-scale thickness of the 2D monolayer enables its conformable attachment with a nearly arbitrary surface. Secondly, 2D monolayers generally possess remarkably high elasticity. The exceptionally high Young's modulus (ranging from 200 to1,000 GPa) and elastic strain limit (up to 20%) empower the 2D monolayer to maintain structural integrity under significant external strain [58]. Based on atomic thickness nanosheets, the sensors can effectively reduce the thickness. Nanoengineering strategies have minimized the thickness of flexible resistive pressure sensors to the sub-micron level, creating a resistive pressure sensor with a thickness of approximately 850 nm, achieving perfect conformal contact with human skin. This design allows for excellent deformation capabilities, achieving an outstanding sensitivity (92.11 kPa⁻^1^) and ultra-low detection limits (< 0.8 Pa) [88].

### Van der Waals Interaction

Beyond their conformability, 2D materials also show enhanced surface stability compared to 3D nanomaterials, owing to their self-saturated surface coordination configuration, which is devoid of dangling bonds [41]. The intralayer van der Waals (vdW) interaction inherent in layered materials not only facilitates the isolation of 2D monolayers [89], but also enables the artificial assembly of various 2D monolayers into heterostructures [66]. This delicate yet potent vdW stacking configuration serves as a versatile tool to finely modulate the optical, electrical, and mechanical properties of heterostructures, promising multifunctionality in ultrathin atomic devices [67, 90]. For instance, van der Waals interaction has been proven to be versatile in tuning the physicochemical properties in the emerging mono-elemental atomic 2D sheets, including structural stability, bandgap engineering, charge carrier injection, flexibility modulation, thereby significantly broadening their applications in energy- and sensor-based nanodevices [91]. In gas sensing, the unique properties of 2D materials make them ideal for chemical detection, offering higher sensitivity and lower detection limits (LODs). Moreover, by combining two or more 2D materials into vdW heterostructures, the synergistic interactions between the nanomaterials can further enhance gas sensing performance. For instance, heterostructured gas sensors comprising a combination of exfoliated MoS₂ and CVD graphene can sensitively detect NO₂ concentrations above 1.2 ppm. Even under mechanical deformation, the performance response characteristics of the sensor remain well preserved [92]. Moreover, despite the covalently self-saturated of 2D materials, the resonant p-electrons form long electron pairs or π conjugation, acting as Lewis bases capable of forming substantial non-covalent adhesion with diverse surface morphologies [93], facilitating conformal integration. Additionally, these electron pairs provide adsorption sites, enabling ultra-high detection limits down to even molecular scale [94].

### Compositional Variety

The burgeoning exploration of 2D monolayers has yielded fruitful achievements ranging from electronic and photonic devices [95], catalysis [96], conductive films [97], mechanical composites [98], and beyond. However, these advancements merely scratch the surface of the vast potential of 2D materials. With the aid of high-throughput computation, it is estimated that over 1825 layered compounds could potentially be readily exfoliated or synthesized [99]. This giant 2D family provides a wealth of opportunities to incorporate a wide array of elements across the periodic table into nanometer-thin flakes, through methods such as direct synthesis of new structures [57] doping with hetero-atoms [100], and controllable extraction of specific element. [101]. Therefore, the exotic properties, facile processability, interfacial compatibility, and component versatility inherent in 2D materials lay a fertile foundation for advancing wearable biodevices and beyond.

## Health Monitoring

**Fig. 3:**
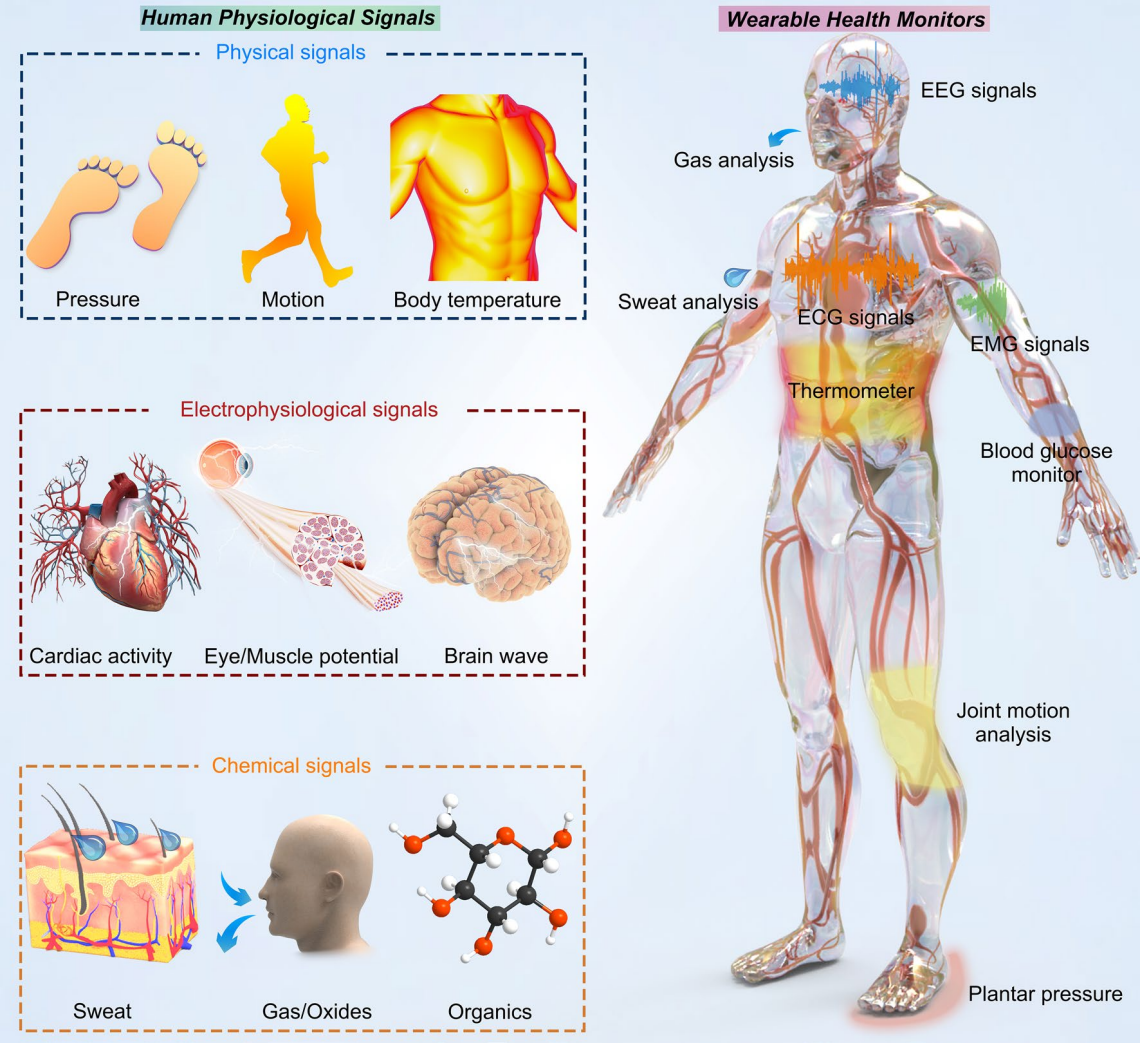
2D materials-based wearable sensors for monitoring physiological signals. 2D material-based wearable sensors for human health monitoring, capable of detecting intricate physiological signals. These sensors are categorized into three main types: physical signals, electrophysiological signals, and chemical signals

Building on the multiple advantages of 2D materials' physical and chemical properties derived from their structures, their potential in health monitoring becomes increasingly evident. In our daily lives, a myriad of biophysical such as mechanical, temperature, and electrophysiological, alongside biochemical indicators like gases and metabolites, are intricately intertwined. These signals hold invaluable insights into the state of health and human performance. Integrated wearable devices have emerged as powerful tools for tracking and analyzing these signals [102–106], thereby enabling real-time health monitoring and fostering bidirectional communication interfaces between humans and machines [107–109]. The continuous evolution of manufacturing technology and the ongoing discovery of new materials have propelled the advancement of wearable devices, offering promising solutions to medical challenges while alleviating financial burdens on healthcare systems. Among the vast emerging materials driving this progress, 2D materials have garnered significant attention due to their atomic thickness, high carrier mobility, and superior flexibility. These unique properties enable the development of wearable devices characterized by lightweight construction, miniaturization, multifunctional integration, enhanced monitoring sensitivity, and expanded monitoring range. In this section, we focus on the latest research breakthroughs in 2D materials-based wearable technology specifically tailored for monitoring health-related human signals **(**Fig. 3**)**.

### Physical Signal Monitoring

Physical signals are crucial in health monitoring, providing insights into the body's condition. Mechanical strains and temperature variations, and other signals carry vital health-related information regardless of their state, whether at rest or during physical activity [110]. The precise detection and prediction of deviations in specific physiological parameters from their normal ranges hold the potential to facilitate more efficient medical interventions, especially in the early stage of disease progression [111]. Consequently, real-time monitoring and dynamic analysis of physical signals are indispensable for recording body movements [112, 113], preventing diseases [114], and managing overall health [115, 116]. As awareness of personal health grows, the demand for wearable or portable sensors has surged. Commercial ventures that integrate real-time motion recording and blood oxygen saturation analysis into portable electronic devices have gained great success [117], reflecting the increasing popularity and utility of such technologies in promoting proactive health management. The integration of advanced materials, such as 2D nanomaterials, further enhances the sensitivity and accuracy of these devices, making them more effective for continuous and non-invasive monitoring in diverse environments. Notably, wearable sensors capable of continuous and real-time monitoring of biohazard gases have attracted increasing research interest and discussion [118]. In this context, 2D materials offer significant advantages, functioning both as direct detectors and as versatile supporting substrates for the design of composite devices with other materials.

#### Mechanical Sensors

By converting mechanical motions produced in human physiological processes into easily measurable electrical data, these sensors reveal widespread applications in multifunctional roles including human health tracking [119, 120]. The remarkable flexibility and huge specific surface area of 2D materials render them capable of withstanding substantial external strains, making them particularly well-suited for applications involving joint motions that demand a wider stretchable range [121]. Furthermore, 2D materials outperform many conventional materials in the precise monitoring of human physiological activities like heartbeat and pulse due to their excellent carrier mobility [122–124].

**Fig. 4:**
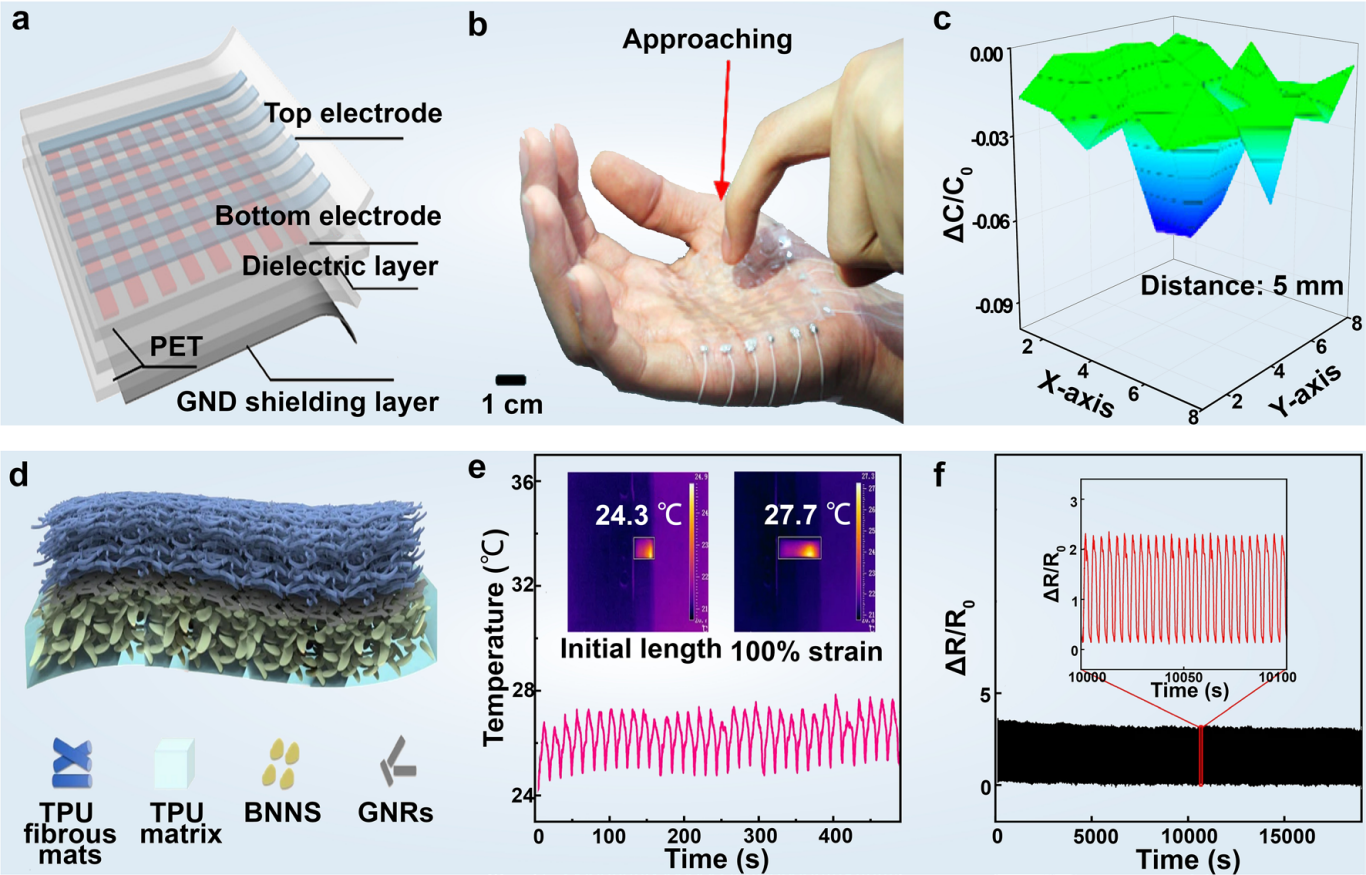
Durable and wide-range mechanical signal detection with 2D material-based wearable devices. **a** Schematic illustrating the concept of a graphene-based capacitive sensor. The sensor consists of three layers: patterned graphene electrodes on a PET film substrate. **b** Photograph of a mutual capacitance touch sensor based on graphene electrodes with a three-dimensional-sensing capability. **c** Capacitance change for approaching finger by 5 mm. Reproduced with permission: Copyright 2017, American Chemical Society [128]. **d** Schematic diagram of the compositional structure of the stretchable resistive strain sensor based on BN nanosheets and graphene nanoribbons (denoted TPU-BNNS). **e** TPU-BNNS strain sensor at the saturated temperature fluctuation range for more than 30 cycles between initial length and 100% strain. **f** Cyclic test of the TPU-BNNS sensor at 100% strain range for more than 5000 cycles, and the inset shows the enlarged view of the marked region. Reproduced with permission: Copyright 2020, Springer Nature [129]

Wearable electronic textiles incorporating graphene exemplify 2D materials that offer scalability, high conductivity, and multifunctionality, particularly in epidermal strain sensing for tracking human joint motion. Compared to conventional wearable sensors, these epidermal sensors provide wireless functionality, lighter weight, larger flexibility, and enhanced compatibility with human skin [125]. Afroj et al. [126] employed a translucent, thin, and stretchable polyurethane-based encapsulant to protect graphene-based wearable e-textiles. This encapsulation layer anchors the conductive graphene flakes to the textile, enabling the repeatable monitoring of finger, wrist, and elbow joint motions with extremely high flexibility and bendability. The textiles exhibit consistent performance in both forward and backward motions, even after home laundry washing cycles. The scalability and multifunctional applications of these highly conductive (sheet resistance ~ 11.92 Ω sq^−1^) graphene-based wearable e-textiles are demonstrated in ultra-flexible supercapacitors and skin-mounted strain sensors. The higher electrical conductivity is achieved by optimizing the average inter-sheet distance of graphene nanosheets through the roller compression and method. This technique effectively reduces the distance, improving the electrical conductivity of graphene-based e-textiles. Multiple treatments significantly lowered the sheet resistance after five padding cycles while preserving the fabric's structural integrity. Beyond composite structures, flat graphene has also revealed high stretchability with remarkable multitouch capability and exceptional sensitivity [127]. Kang et al. [128] developed an auxetic mesh graphene-based touch sensor, featuring doped graphene electrodes with a conductivity of 317.1 Ω sq^−1^ and optical transmittance exceeding 84.6%. Elevated sensitivity arises from using modeling software to regulate the spacing between graphene electrodes and the distribution of the elastic modulus in each layer of the device, ensuring the stability of the electrical performance before and after bending. The perforated graphene structure facilitates conformal contact with highly deformable human body parts such as forearms and palms, allowing for multiple touch signal detection, acute recognition of the distance and shape of approaching objects, and the construction of assistive communication devices (Fig. 4a–c).

Due to the detrimental effects on performance, lifespan, and reliability, continuous mechanical stimuli-induced massive heat emission poses a longstanding challenge in real-time monitoring devices, particularly in widely studied resistive sensors. In this regard, Tan et al. [129] proposed a hierarchical wearable strain sensor composing a thermoplastic polyurethane (TPU)-based fibrous membrane (denoted TPU-BNNS), a network of graphene nanoribbons, and a boron nitride (BN)-filled TPU matrix. The TPU-encapsulated graphene serves as a precise sensor for human motion, while the TPU-BN matrix ensures rapid dissipation of operational heat during dynamic monitoring. The thermal stability arises from the innovative interface design: when the device returns to its original length after being stretched, effective bridging between boron nitride nanosheets increases the contact area, aiming to reduce thermal resistance in the nanocomposite and providing efficient thermal pathways. Therefore, this wearable strain sensor shows a remarkable 242% enhancement in thermal conductivity and realizes impressive thermal management, maintaining equilibrium temperature variation within 3.5 °C after undergoing continuous stretching-releasing cycles for 30 cycles. Moreover, the sensor demonstrates excellent stability and repeatability over 5,000 cycles at 100% strain (Fig. 4d–f). Notably, recent advances in hexagonal boron nitride show that it can offer more than its conventional role as an insulating or thermal-conductive layer. Its emerging electronic and photonic properties present intriguing possibilities for inducing additional functionalities in the design of low-energy electronics [130].

In addition, apart from enhancing strain sensors through efficient heat dissipation, preparing strain sensors capable of functioning across a wide temperature range proves to be an effective strategy. Xie et al. [131] utilized MXene nanosheets and polyetherimide fibers to prepare strain sensors capable of operating from −5 °C (with a sensitivity of 80 kPa^−1^) to 150 °C. These piezoresistive strain sensors display ultra-high sensitivity (20 kPa^−1^), a low detection limit of 9 Pa, and a rapid response time of 163 ms. They also demonstrated excellent durability over 10,000 cycles at room temperature, 2,000 cycles at 100 °C, and 500 cycles at −5 °C. Moreover, the sensor exhibits sensitive responsiveness to external mechanical stimuli spanning a wide temperature ranging from 150 °C to liquid nitrogen. The superior sensitivity of the device across a wide temperature range results from the combination of excellent mechanical properties and dimensional stability of PEI, ensuring reliable performance under varying temperature conditions. Furthermore, the metallic conductivity and hydrophilicity of MXenes facilitate effective signal transduction, further enhancing sensitivity in both low and high-temperature environments. In addition, the fiber network shows excellent Joule thermal properties, reaching 78 °C at an applied voltage of 12 V. Thus, the modified sensor holds considerable potential for applications in wearable clothing and personal heating systems operating in harsh temperature conditions.

**Fig. 5:**
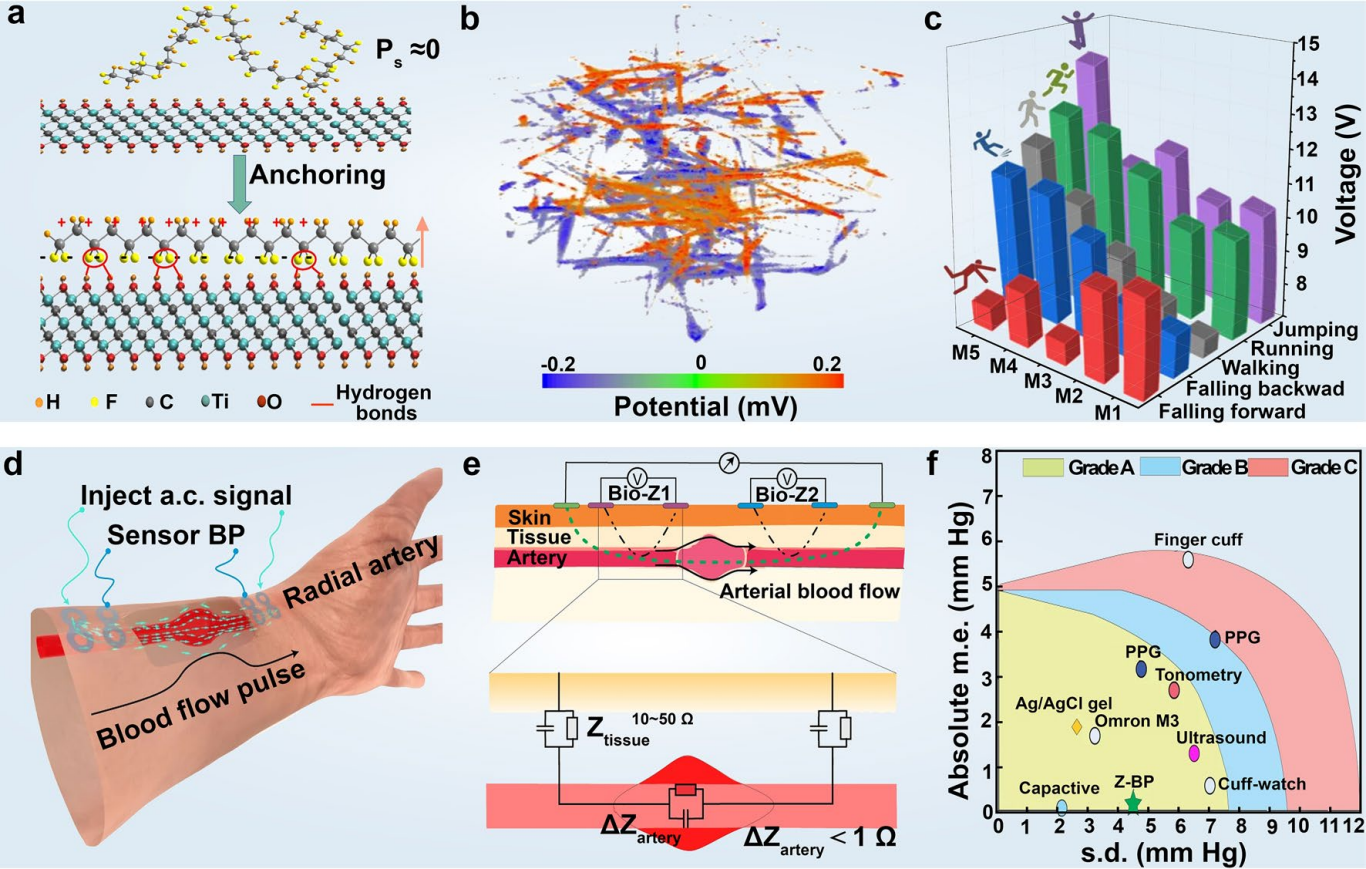
High-sensitivity and dynamic monitoring of mechanical signals with 2D material-based wearable devices. **a** Schematic representation of in situ stretching and alignment of PVDF polymer chains to enhance spontaneous polarization (P_s_) by the surface termination of Ti_3_C_2_T_x_ nanosheets. **b** Piezoelectric potential distribution in the visual structural modeling of the prepared PVDF and MXene composite textile. Note: For simulation, the stress applied along the z-axis is fixed at 10^6^ Pa. **c** MXene-based piezoelectric sensor for gait monitoring. Reproduced with permission: Copyright 2022, Springer Nature [132]. **d** Schematic diagram of graphene electronic tattoo for blood pressure detection. **e** Schematic diagram of the blood pressure detection principle. **f** Comparison of the detection performance of graphene electronic tattoo with that of the commercial blood pressure detection devices rated as class A. Reproduced with permission: Copyright 2022, Springer Nature [133]

Polyvinylidene fluoride (PVDF) plays a critical role in wearable piezoelectric sensors owing to its lightweight nature, flexibility, biocompatibility, and ease of processability. Among the various crystalline phases in PVDF, β-phase, characterized by all-trans configuration and strong polarization, is highly desirable for its excellent piezoelectricity. Su et al. [132] reported that the surface hydroxyl functional groups on Ti_3_C_2_T_x_ MXene enabled the dipole alignment and enhanced the net spontaneous polarization of polymer-ceramic composites. PFM characterization along with phase-field simulations and MD calculations, provides a comprehensive analysis revealing that strong hydrogen bonding between MXene nanosheets and hydroxyl terminals on -CF facilitates the long-term growth of the all-trans conformation and the alignment of domain directions, thereby improving the $\beta$-phase content and piezoelectric coefficient. This enhancement resulted in a piezoelectric response increase of up to 160% in PVDF composites, making them readily integrable into shoe insoles for comprehensive gait patterns monitoring, identification of walking habits, and prognosis of conditions like Metatarsalgia prognosis (Fig. 5a–c).

In wearable strain sensors, there generally exists a dilemma between high sensitivity and a wide strain range. Achieving both simultaneously poses a challenge because high sensitivity requires significant structural variation under minor strain, whereas maintaining the connectivity of the connectivity of conductive pathways is necessary under large deformations. To address this dilemma, Yang et al. [134] constructed a full-range human motion sensor with a hybrid network structure using MXene nanoparticles and nanosheets. The migration of nanoparticles contributes to resistance variation against external mechanical stimuli, while the wrapping effect of 2D sheets bridges detached nanoparticles to maintain connectivity across a broad strain range. This synergetic motion mode of MXene endows the hybrid network with a high gauge factor (> 178.4) over the entire broad range (53%), an ultralow detection limit (0.025%), and good durability over 5,000 cycles. Additionally, Xu et al. [135] developed a covalent strategy to enhance the interlayer strength of 2D MXene flakes, further increasing the detection range up to 200% strain with an acute response time of 70 ms.

Dynamic monitoring of arterial blood pressure in non-clinical settings is essential for comprehensively assessing daily health conditions, diagnosing medical issues, identifying root causes, and proactively preventing diseases. The atomic thickness, self-adhesion, lightweight, and electrical conductivity of 2D materials endowed 2D material-based devices with comfort and noninvasiveness compared to conventional ambulatory sensors. Kireev et al. [133] introduced graphene electronic tattoos for continuous blood monitoring via electrical bioimpedance. The intimate contact of the graphene tattoo with the skin results in remarkably high accuracy, with diastolic pressures exhibiting 0.2 ± 4.5 mm Hg and systolic pressures of 0.2 ± 5.8 mm Hg, ranking the tattoo sensor among Grade A wearable blood pressure measuring devices (Fig. 5d–f). The high precision of the graphene tattoos in blood pressure monitoring relies on the CVD prepared graphene-based materials, which provides large-area uniformity and reliable electrical performance.

#### Temperature Sensors

Body temperature represents a crucial parameter for diagnosing many diseases, with a continuous temperature profile offering vital insights into disease diagnosis and treatment strategy [136–138]. Despite its significance, monitoring skin temperature poses challenges due to its susceptibility to environmental influences, resulting in a scarcity of wearable temperature devices on the market. Real-time health monitoring necessitates temperature change responsiveness as an essential feature of intelligent wearable devices. Taking advantage of the thermal response properties and excellent carrier transport capabilities of 2D materials, wearable sensors can achieve reliable and sensitive body temperature monitoring, thus significantly enhancing human health monitoring capability [139].

To achieve a wide range of temperature responses and excellent environmental stability, Chen et al. [140] used GO, rGO, and PVA hydrogel to prepare a multi-function sensor with a high temperature coefficient resistance (TCR = 3.81 °C^−1^). The sensor exhibited reliable mechanical and electronic properties across a wide temperature range (30 ~ 60 °C), showcasing excellent mechanical strength, toughness, and environmental stability. On one hand, DMSO in the organohydrogels sensor can influence the interactions with polymer chains during the freeze polymerization process, imparting antifreeze properties and long-term environmental stability to the sensor. On the other hand, the GO/rGO conductive nanofillers with rich functional groups promote the formation of structurally intact regular closed pores in the organohydrogels, resulting in enhanced mechanical properties. Alternatively, while graphene grown via CVD is ultrathin and transparent, its direct usage as sensor materials generally reveals some intrinsic drawbacks such as low sensitivity, complex processing, and low scalability. In this context, Shirhatti et al. [141] employed graphene ink and a laser etching process to prepare an ultra-thin interdigital electrode with high sensing performance. This sensor demonstrated outstanding temperature response and monitoring stability, enabling continuous tracking of minute variations in body temperature.

**Fig. 6:**
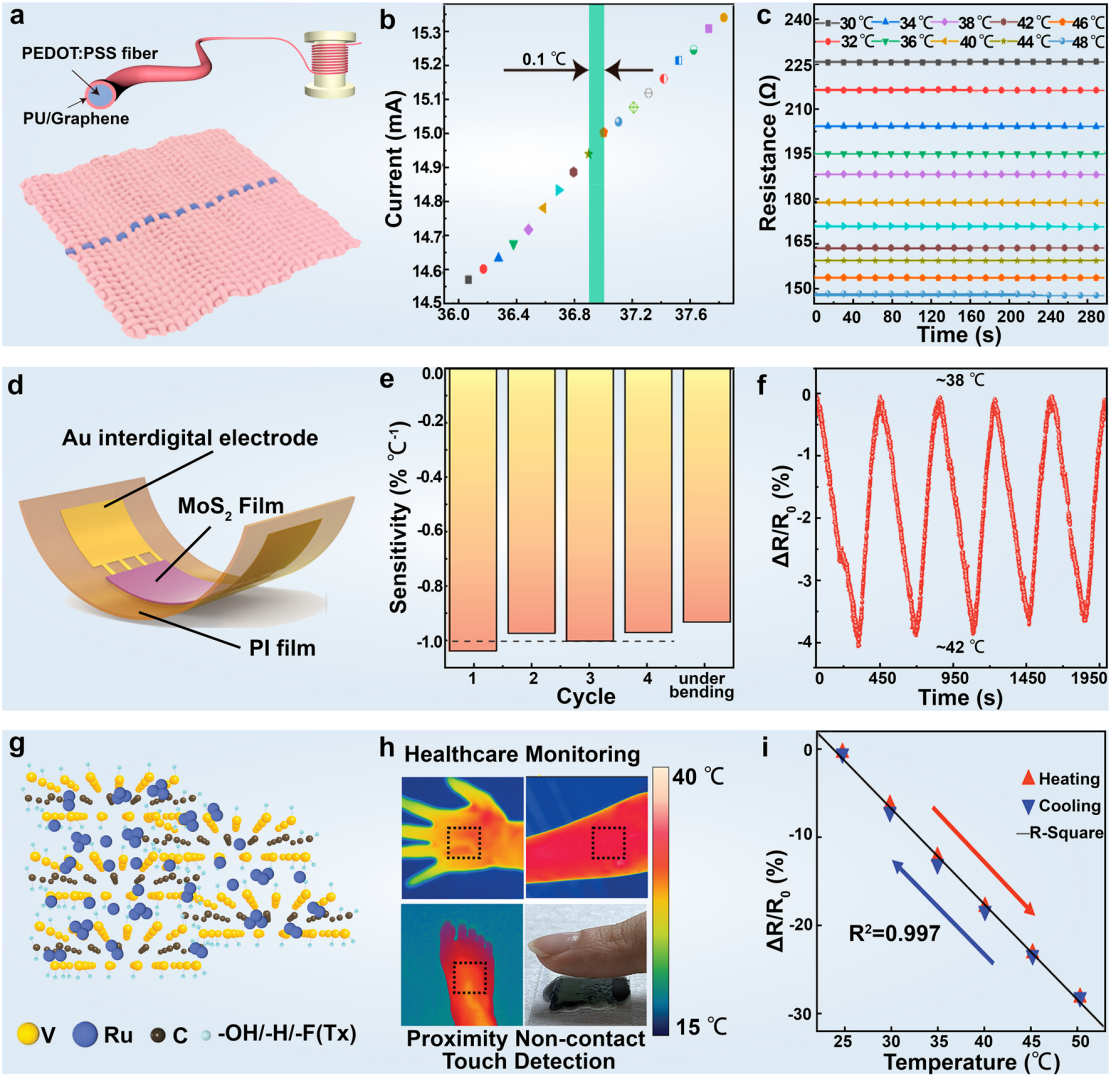
2D material-based wearable devices for body temperature detection. **a** Schematic representation of PU/graphene encapsulated skin–core structure PEDOT: PSS and composite fibers prepared into fabrics. **b** Current profiles of the composite fibers within the temperature range of 36.1–37.8 °C with an increment of 0.1 °C. **c** Stability of resistance exhibited by the composite fibers at different temperatures. Reproduced with permission: Copyright 2023, American Chemical Society [142]. **d** Structural schematic of the MoS_2_-based temperature sensor. **e** Calculated sensitivity of the MoS_2_ temperature sensors across various heating cycles and in the folded state, displaying an averaged sensitivity of -0.98 ± 0.03% °C^−1^. The sensitivity is determined using the (R_1_-R_0_)/R_0_(T_1_-T_0_) equation, where R_0_ and R_1_ represent the resistance at temperatures T_0_ and T_1_, respectively. **f** Dynamic cyclic electrical response and recovery curves of the sensor measured within the range of 38–42 °C. Reproduced with permission: Copyright 2022, Wiley–VCH [143]. **g** Schematic illustration of the heterojunction structure formed through the combination of Ru with V-MXene via atomic layer deposition. **h** Schematic depiction of the temperature sensor achieving both contact and non-contact temperature sensing. **i** Relative resistance changes of the developed temperature sensor as a function of temperature. Reproduced with permission: Copyright 2023, Wiley–VCH [144]

For the fabrication of stretchable and highly sensitive temperature sensors capable of withstanding body strain, Trung et al. [145] developed transparently stretchable temperature sensors using rGO nanosheets and elastic TPU. These TS-gated temperature sensors showed high sensitivity and maintained their response after enduring ten thousand stretching cycles, enabling straightforward skin temperature monitoring. However, conventional flexible temperature sensors, typically constructed on impermeable substrates like PDMS and PET [146, 147], often lead to sweat accumulation and potential skin inflammation during prolonged attachment to the skin [148, 149]. In this regard, Fan et al. [142] devised a skin–core structure polyurethane (PU)/graphene encapsulated poly(3,4-ethylenedioxythiophene) − poly(styrenesulfonate) (PE-DOT:PSS) temperature-sensitive fiber via wet spinning and impregnation technologies. The composite fiber showed high sensitivity (− 1.72% °C^−1^), super-resolution (0.1 °C), fast time response (17 s), anti-sweat interference, and high linearity (R^2^ = 0.98), facilitating real-time body temperature monitoring between 30 − 50 °C without interruption during daily activity when integrated into comfortable and durable fabrics. Through Bluetooth wireless transmission, real-time body temperature data can be conveniently displayed on mobile phones for parents or guardians (Fig. 6a–c). Furthermore, Ryu et al. [150] reported a reduced graphene oxide-based sensor compatible with daily clothing and gloves, revealing excellent sensitivity (-0.285% °C^−1^), rapid response (11.6 s), and recovery (14.8 s) times within the temperature range of 25 ~ 45 °C.

The linear relationship between temperature and resistance is crucial for the development of high-performance temperature sensors. Taking advantage of the high sensitivity of MoS_2_ to thermal fluctuations, Li et al. [143] utilized printed and annealed MoS_2_ films to fabricate temperature sensors. Demonstrating a linear decrease in resistance with rising temperature, these sensors exhibited high sensitivity and swift linear response across the entire temperature range. Upon attachment to the skin, the sensors accurately recorded surface temperature variations, affirming their excellent sensing performance. In addition, the sensor showcased outstanding mechanical flexibility, durability, and stability approximately 2% resistance variation over 10,000 bending cycles (Fig. 6d–f).

MXene possesses numerous excellent properties, such as electrical conductivity and bending strength comparable to that of graphene. Therefore, as an emerging new material, MXene has gradually gained more attention in flexible electronics. Zhang et al. [151] prepared a sensor using Ti_3_C_2_T_x_, 3,4-ethylene dioxythiophene, and highly elastic polystyrene block polymers, capable of sensitively monitoring body temperature. This sensor enables seamless contact with the human body for enhanced comfort with a high-temperature coefficient of resistance, enabling real-time monitoring of human body temperature, thus holding promise for smart skin and healthcare applications. To address the persistent issue of oxidation in MXene, Luo et al. [152] used MXene-modified polydopamine (PDA) to enhance the waterproof and breathable properties of wearable sensors. Incorporating polydimethylsiloxane (PDMS) packaging, the designed temperature sensor exhibited excellent hydrophobicity and air permeability, ensuring stable sensing performance and enduring temperature monitoring performance. Moreover, the V-MXene offers a substantial active area per mass and lighter layered components compared to widely explored Ti-MXenes. These properties, coupled with ideal electron transport channels, result in rapid charge-carrier transfer, surpassing other 2D materials such as graphene, MoS_2_, and boron nitride [153, 154]. Mohapatra et al. [144] further enhanced the electronic properties and electron transport channels of V-MXene by modifying it with Ru, yielding high-performance temperature sensors capable of contact and non-contact temperature monitoring. These sensors hold broad prospects in mobile medical and non-contact man–machine interface applications (Fig. 6g–i) (Table 1).

**Table 1:** Wearable devices utilizing 2D materials for strain and temperature sensing

Sensor type	Substrate	2D materials	Maximum stretchability	Sensitivities	Durability (cycles)	Applications	References
Strain sensor	PDMS	Ti_3_C_2_	~ 53%	GF > 178.4	> 5000	Speech recognition	[134]
	TPU	Ti_3_C_2_-rGO	~ 200%	GF ~ 84,326	> 5000	Gesture recognition	[135]
	PET	Graphene	~ 15%	-	-	Touch sensor	[128]
	TPU	Graphene	~ 100%	GF ~ 35.7	> 15,000	Motion monitoring	[129]
	PEI	Ti_3_C_2_	-	80 kPa^−1^	> 10,000	Motion monitoring	[131]
	PVA	Ti_3_C_2_	> 900%	GF > 14,000	> 10,000	Motion monitoring	[104]
	Cellulose	Ti_3_C_2_	~ 95%	114.6 kPa^−1^	> 10,000	Motion monitoring	[123]
	PI/PDMS	Ti_3_C_2_	-	24.63 kPa^−1^	> 5000	Pulse monitoring	[155]
	Silk fibroin	Ti_3_C_2_	-	298.4 kPa^−1^	> 10,000	Heartbeat monitoring	[156]
	PVA	GO	~ 80%	10.0 kPa^−1^	> 100,000	Motion monitoring	[157]
	Chitosan	Ti_3_C_2_	~ 99%	80.4 kPa^−1^	> 150,000	Motion monitoring	[158]
Temperature sensor	PU	rGO	~ 70%	~ 1.34% ºC^−1^	> 10,000	Body temperature monitoring	[145]
	Ecoflex	Graphene	~ 800%	-	> 1000	Body temperature monitoring	[159]
	PDMS	Graphene	~ 36%	~ 7.04 × 10^–5^ ºC^−1^	> 500	Body temperature monitoring	[160]
	PI	Graphene	-	~ 0.83% ºC^−1^	> 20,000	Body temperature monitoring	[161]
	PI	MoS_2_	~ 1.25%	~ 0.98% ºC^−1^	> 10,000	Body temperature monitoring	[143]
	PU	Graphene	~ 15.19%	~ 1.72% ºC^−1^	> 3000	Body temperature monitoring	[142]
	Ru	V-MXene	-	~ 0.42% ºC^−1^	-	Body temperature monitoring	[144]
	SEBS	Ti_3_C_2_	~ 22%	~ 0.86% ºC^−1^	> 20,000	Body temperature monitoring	[151]
	PVA	Graphene	~ 660%	~ 0.38% ºC^−1^	-	Body temperature monitoring	[140]

Note: GF stands for Gauge Factor

### Electrophysiological Signal Monitoring

Beyond physical signals, electrophysiological signals offer a window into the body's internal functions, monitoring subtle changes in electrical potential and impedance that are closely related to human activities and serve as key indicators of health status. In response to external stimuli, the activation of neurons and associated electrical pathways generates various electrophysiological signals, mainly encompassing electrocardiogram (ECG), electroencephalogram (EEG), electromyogram (EMG), and electrooculogram (EOG), etc. [162]. Considering the close relationship between organ, tissue, and neuronal activities with electrical potential [163], these electrophysiological signals serve as valuable indicators of human health status, and their temporal logging and analysis are crucial for health monitoring [164, 165]. The conventional use of rigid electrodes and gel-based 12-lead electrocardiogram databases represents a standard clinical procedure for noninvasive arrhythmias and other heart disease diagnoses [166]. However, this approach requires several improvements, for instance, the gel used can be irritating to the skin, and its tendency to dry out hampers the long-term and stable measurements [167, 168]. Moreover, the use of multiple gel electrodes for multichannel electrophysiology involves multiple lead wires and snap buttons [169], significantly reducing comfort, mobility, and signal quality, particularly under motion [170]. Researchers have thus been exploring more convenient and reliable systems and methods for daily health monitoring, noninvasive detection, and early disease diagnosis [171, 172]. Ultrathin, flexible, and self-adhesive devices based on 2D materials have emerged as promising alternatives, offering significantly enhanced skin fit and comfort. In addition, 2D materials like MXene and metallic flakes exhibit low signal-to-noise ratios and high monitoring sensitivity due to their superior electrical properties and outstanding interfacial contact.

The electrical impedance of the electrode − skin interface can be presented by a complex expression ${\text{Z}}(\omega ) = {\text{R}}/(1 + {\text{j}}\omega {\text{CR}})$ in RC-circuit models, where *R* and *C* denote the resistance and capacitance of the skin layer, *ω* represents angular frequency, and *j* is the imaginary unit. The magnitude and stability of e-skin impedance significantly impact the quality of electrical recordings. High and unstable impedance can lead to poor signal quality. In systems with multiple electrodes, elevated interelectrode impedance also diminishes the effectiveness of common mode noise rejection, as it amplifies differences between electrodes. Carefully chosen geometries and material compositions of soft, skin-like electrodes facilitate irritation-free, conformal contact with the skin and establish a low-impedance measurement interface [167, 173–176].

**Fig. 7:**
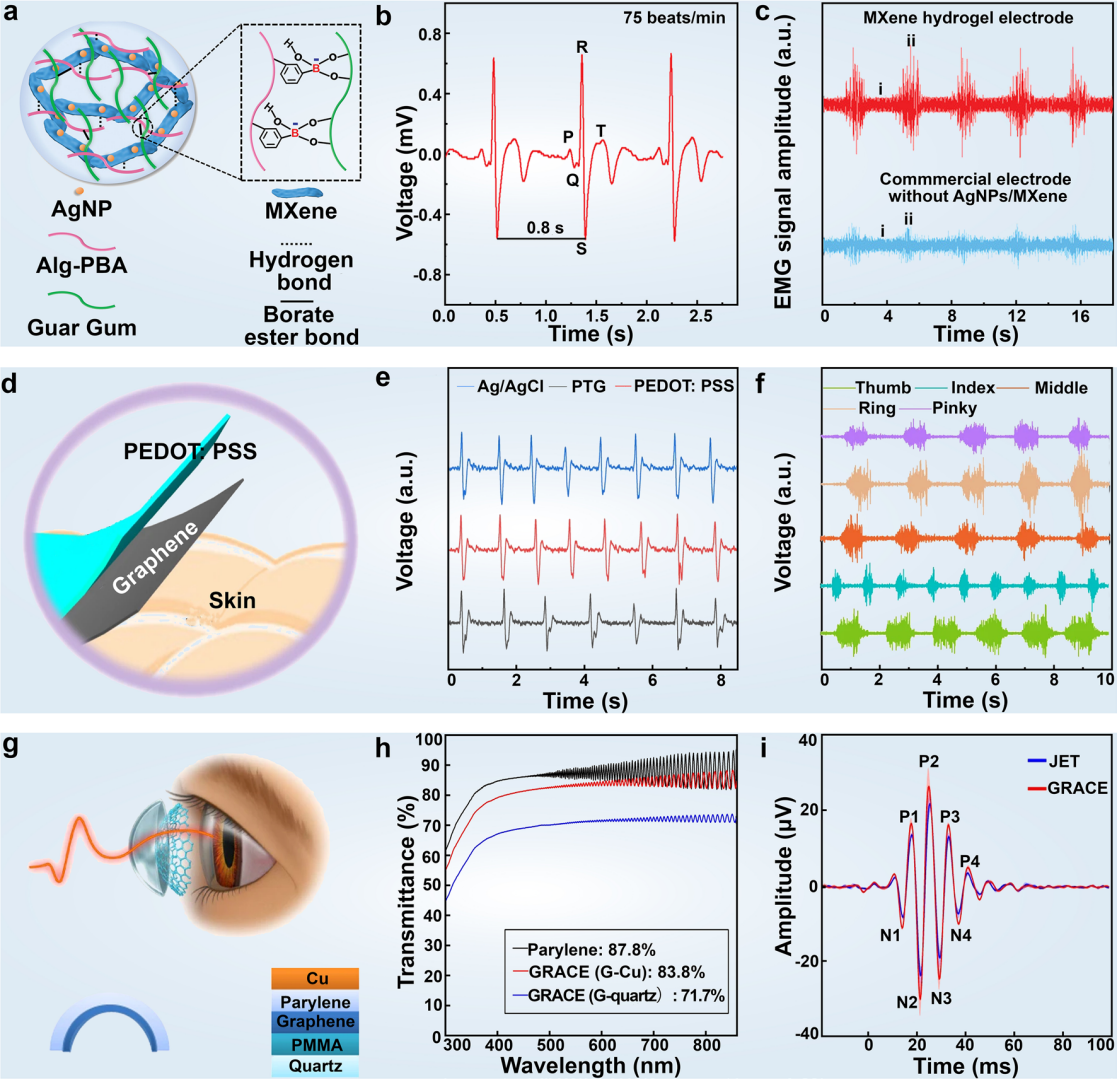
Detection of electrophysiological signals with 2D material-based wearable devices. **a** Schematic structural diagram of the MXene hydrogel epidermal sensor. **b** The epidermal sensor assembled from MXene hydrogel for ECG detection. **c** Comparison of EMG detection performance between MXene-based epidermal and commercial sensors. Reproduced with permission: Copyright 2022, Wiley–VCH [177].** d** Schematic structural diagram of graphene-based electronic skin. **e** EOG measured with PTG (red), pure PEDOT: PSS (blue), and Ag/AgCl (black) electrodes showing peaks and troughs corresponding to eyelid movements. **f** PTG acquires sEMG from finger movements (thumb, index finger, middle finger, ring finger, and little finger). Reproduced with permission: Copyright 2021, Springer Nature [178]. **g** Structural diagram of graphene-based contact lens for ERG monitoring. **h** Optical transmittance of the bare Parylene-C, and graphene contact lens electrodes (GRACE) devices made from graphene grown on quartz (G-quartz) and graphene grown on Cu (G-Cu), all with Parylene thickness of 25 μm. The transmittance at 550 nm wavelength is shown in the inset. **i** Comparison of ERG monitoring performance with commercial electrodes. Reproduced with permission: Copyright 2018, Springer Nature [179]

Researchers have been actively seeking a convenient and reliable new system and method for daily health monitoring, noninvasive detection, and early disease diagnosis [171, 172]. One of the primary challenges is accurately detecting weak electrophysiological signals, some as low as micro-volt scale [178], particularly for reliable measurement amidst human movement. Taking advantage of the negatively charged hydrophilic surfaces, large specific surface area, and high electrical conductivity, Li et al. [177] fabricated a MXene-based epidermal sensor. These devices exhibit high-quality ECG signals, including distinguishable P-wave (atrial depolarization), QRS complex (ventricular depolarization), and T-wave (ventricular repolarization), crucial for diagnosing cardiovascular-related diseases [28]. Additionally, incorporating an extra MXene-based electrode on the other forearm effectively mitigates the influence of human movement on EMG signal detection, resulting in an impressively higher signal-to-noise ratio of 17.80 dB compared to commercial counterparts of 10.17 dB. With its good conformability, high conductivity, and outstanding dynamic sensing performance, the MXene-based sensor holds great promise for applications like postoperative rehabilitation training, muscle damage treatment, and the diagnosis of muscle- and cardiovascular-related diseases (Fig. 7a–c).

Due to its ultralow weight and thickness, excellent optical transparency, high electrical conductivity, outstanding mechanical properties, and electrochemical stability, graphene has been regarded as a prime candidate for skin electrodes in electrophysiological signal detection [58, 166, 180–184]. Das et al. [185] developed a flexible and conductive chemically reduced graphene oxide-based electrode for uninterrupted long-term ECG monitoring without allergic reactions. Moreover, Das et al. [186] fabricated a high-performance paper-based epidermal sensor using thermally reduced graphene oxide and nylon film (TRGO/NM), exhibiting high sensitivity (40 Ω sq^−1^) and fast response time (~ 0.5 s), suitable for ECG, EEG, and EMG measurements of electrophysiological signals without cytotoxicity concerns associated with conventional Ag/AgCl sensors. Zhao et al. [178] developed an ultrathin (~ 100 nm) dry epidermal electrode based on CVD-grown graphene. Specifically, the PEDOT-PSS polymer layer not only assists the conformal transfer of graphene onto various tissue surfaces for gel-free measurements but also injects appreciable charge concentration into the graphene layer (total sheet resistance down to ~ 24 Ω sq^−1^) due to the strong interfacial π-π interaction. The thin-film electrodes demonstrated good recording capabilities of electrophysiological signals, such as face skin and brain activity during motion (Fig. 7d–f), enabling human–computer interaction and long-term monitoring of mental and physical health. However, CVD-grown graphene faces challenges under strain [187, 188], prompting research into various patterning techniques, including wrinkling-engineering  [189], kirigami/origami technology [190], printing/nanofabrication [191], etc., for enhanced flexibility of graphene [192] as well as many other nanomaterials [193]. By virtue of the excellent flexibility of porous PDMS substrate, Yun et al. [194] achieved superior stretchability (up to 150% strain) in graphene-based bioelectrodes by coating and patterning on a porous PDMS substrate. The ECG, EEG, and EMG measurements via graphene-based dry electrodes resemble the signal quality obtained simultaneously via commercial Ag/AgCl-gel electrodes. Additionally, Wei et al. [195] showed the multifunctional integration of graphene-based textiles using laser scribing and thermal transfer technology. The firm attachment between laser-induced graphene with textiles enables multifunction integration, including strain sensing, pressure sensing, physiological electrical sensing, and sound generation, offering a non-disruptive warning system for abnormal physical states via the thermoacoustic effect, thus avoiding discomfort caused for users.

The broad interest in eye health has recently spurred appreciable research efforts into wearable sensors suitable for ocular electrophysiology. Du et al. [196] designed a novel graphene structure intercalated with molybdenum chloride (MoCl_5_) in the van der Waals gap of graphene (denoted as Mo-FLG), where MoCl_5_ serves as both a lubricant to weaken interlayer interactions and an electrical crack connector to bridge cracked domains at high strain. The Mo-FLG electrode exhibits excellent capabilities in detecting electrophysiological signals, including EMG, ECG, and EEG as aforementioned, as well as electrooculogram monitoring, with high single-to-noise ratios comparable to commercial Ag/AgCl electrodes. Visual electroretinography measurement (ERG) plays a crucial role in ophthalmic diagnostic tests for assessing the functional integrity of the retina. Wearable sensors with exceptional optical transparency and softness are indispensable for ERG measurements with high signal amplitude and stability. Yin et al. [179] reported the fabrication of soft graphene contact lens electrodes with broad-spectrum optical transparency, showcasing their application for in vivo visual electrophysiology measurements. The combined metrics of graphene including softness, lightweight, conformal adhesion against eye movements, and full-cornea recording capability, enhance the integrated ERG test performance, surmounting clinically used ERG-Jet electrode (Fig. 7g–i) (Table 2).

**Table 2:** Summary of wearable devices based on 2D materials for electrophysiological monitoring

Substrate	2D materials	Signal	Measure locations	Measurement signal	References
Silicone elastomer	Graphene	Voltage	Forehead, chest, forearm, face, neck, finger	EEG, ECG, EMG	[197]
PAA	MXene	Voltage	Finger, throat, elbow joint, wrist	EEG, ECG	[198]
PI	Graphene	Voltage, resistance	Chest, forearm	ECG, EMG	[199]
TPU	MXene	Voltage	Arm	ECG, EMG	[200]
PI	Graphene	Voltage	Elbow, chest	EEG, ECG, EMG	[164]
PI	Au nanosheets	Voltage	Elbow	ECG, EMG	[167]
PDMS	Graphene	Voltage	Skin	ECG	[171]
PVA	MXene/GO	Voltage	Arm, neck	ECG, EMG	[172]
Guar gum	MXene	Voltage	Arm	ECG, EMG	[177]
PEDOT:PSS	Graphene	Voltage	Forehead, finger, face, arm	EMG, ECG, EOG, EEG	[178]
Parylene-C	Graphene	Voltage	Eyes	ERG	[179]
Nylon	GO	Voltage	Forehead, arm, finger	EMG, ECG, EEG	[186]
PDMS	rGO	Voltage	Arm, leg, forehead, knee, finger	EMG, ECG, EEG	[194]
SEBS	Mo-BLG	Voltage	Forehead, hand, leg, arm, cheekbone, neck	EMG, ECG, EOG, EEG	[196]
TPU	Au nanosheets	Voltage	Heart, leg, nerve (animal experiments)	ECG, EMG, sciatic nerve signals	[201]
PA66	Graphene	Voltage, resistance	Finger, throat, chest	ECG	[14]
PAAm	Sulfonated GO	Voltage	Wrist, forehead, neck, back, muscle (animal experiments)	EMG, ECG, EEG	[202]
PU	Au nanosheets	Voltage, current	Wrist, finger, leg	ECG	[203]
PU	MXene	Voltage, resistance	Wrist, arm, throat, finger, chest	ECG, EMG	[204]
PAA	MXene	Voltage	Forehead, arm,	EMG, ECG, EOG	[205]
PDMS	Au nanosheets	Voltage	Arm, forearm, chest	ECG, EMG	[206]

### Chemical Signals Monitoring

Electrophysiological signals reveal electrical activity in the body, while chemical signals are key indicators for bio-diagnosis, health management, and environmental conditions, offering complementary insights into physiological and environmental states. The past few decades have witnessed significantly increased consciousness regarding health conditions, leading to a broad research interest in chemical sensors for environmental and health monitoring [207–209]. Researchers have expanded their focus beyond tracking physical exercise activity, directing substantial efforts toward incorporating chemical detection capabilities into flexible devices. Specifically, various toxic gases associated with inhalation [210, 211], such as carbon/nitrogen/sulfur oxides, respiratory irritants, volatile organic compounds, and more, have been identified as causes of pulmonary injury, systemic diseases, and other health issues [212, 213]. Additionally, human expired gas comprises a complex mixture of more than 3,000 components, many of which are present in deficient concentrations (part per million scale) yet provide abundant information for the early-stage diagnosis of subhealth or diseases [214, 215]. The exceptional distinguishability down to the single molecule level in 2D materials [94, 216] renders them competitive candidates for developing ultra-sensitive chemical sensors [37, 42, 56, 217–219]. Therefore, wearable gas sensors based on 2D materials have shown remarkable advantages in the rapid and sensitive monitoring of harmful gases in the environment, early diagnosis, and timely warning of diseases, thereby playing crucial roles in personal health protection and diagnosis [220] (Table 3).

**Table 3:** Summary of main characteristics of 2D material gas detectors

2D materials	Analytes	OT (℃)*	LODs (ppm)*	t_res_/t_rec_ [s]*	References
GO	NH_3_	RT	1.5	68/274	[239]
SnO_2_	CH_4_, C_2_H_4_, C_2_H_6_, C_3_H_6_, C_3_H_8_, 1-C_4_H_8_, n-C_4_H_10_	300	-	-/-	[240]
rGO	NO_2_	RT	0.0435	140/630	[229]
Graphene	CH_3_COCH_3_	RT	37–167	10/10	[241]
rGO	NO_2_	RT	-	114/2400	[242]
rGO	NO_2_	RT	0.2	220/-	[243]
WS_2_	RH	RT	20 (%RH)	5/6	[244]
RuO_2_	NO_2_	80.3	5	-/-	[230]
MoS_2_	NO_2_	RT	-	-/-	[245]
rGO	H_2_S, C_2_H_5_OH, H_2_	-	1–5	-/-	[246]
rGO	NO_2_	RT	-	-/-	[247]
NbWO_6_	H_2_S	150	-	6/30	[208]
Graphene	NO_2_	RT	1	89/579	[248]
rGO	NO_2_	RT	-	-/-	[249]
rGO	NH_3_	RT	-	1200/5400	[250]
NbSe_2_	NO_2_, NH_3_	RT	0.12	-/-	[251]
Graphene	NO_2_	RT	0.0012	360/720	[252]
Graphene	NO_2_	RT	0.013	7/22	[253]

^*^OT Operating temperature, *LODs* Lower detection limits, *t*_*res*_ Response time, *t*_*rec*_ Recovery time

#### Chemical Signals in Gases

The rapid advancement of industrialization and urbanization has exacerbated environmental degradation, especially concerning gaseous pollutants. Inorganic compounds like NO_2_, NO, and NH_3_, along with organic compounds such as methanol, acetone, etc., are recognized as toxic environmental gases and biomarkers for certain diseases [213, 224–226]. To address the urgent need for timely detection of these hazardous gases, mitigate respiration-caused physiological harm, and facilitate disease diagnosis, there is a growing emphasis on the development of low-cost, durable, and high-performance gas sensors in current research endeavors [209, 218, 227, 228].

**Fig. 8:**
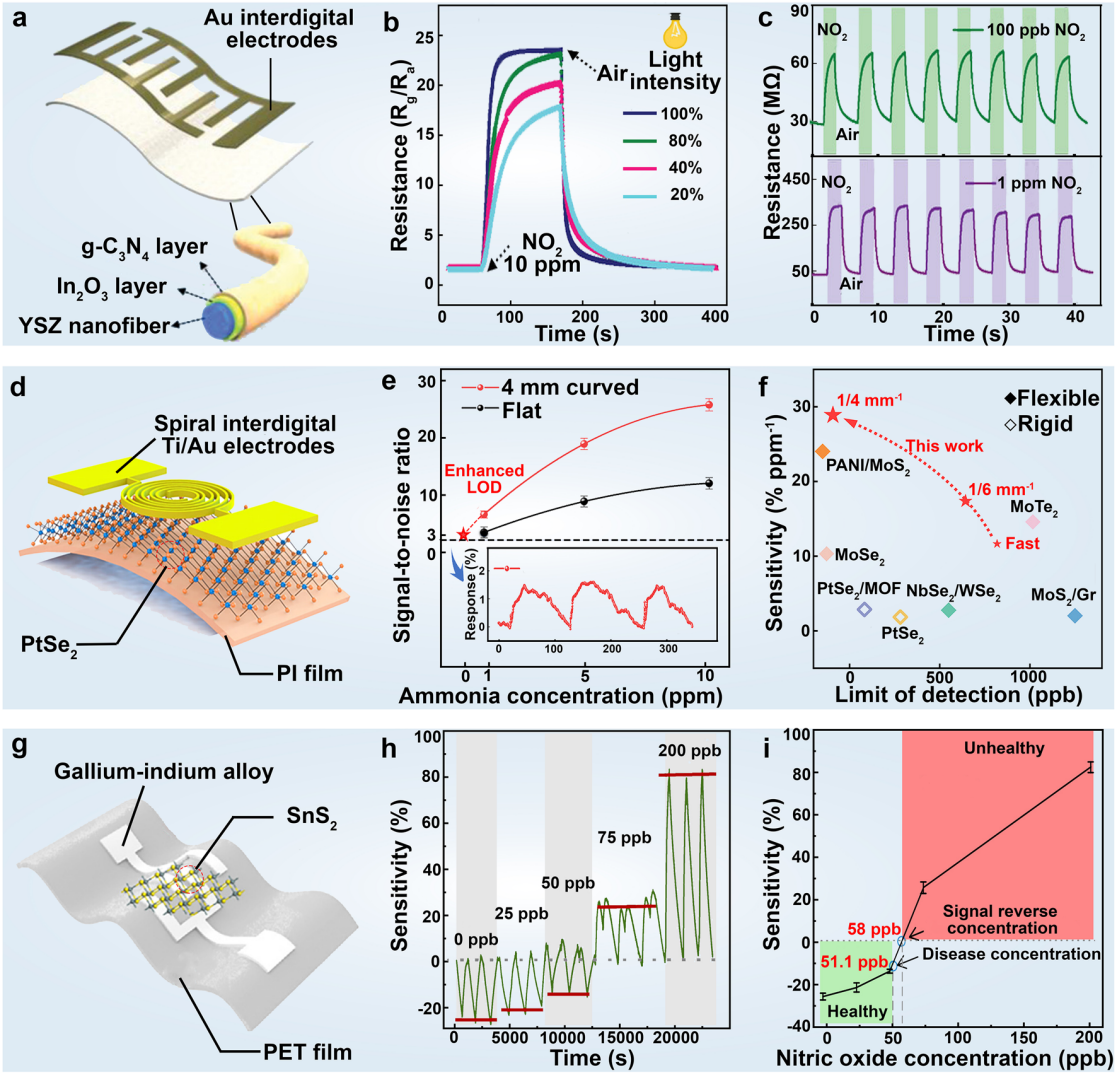
Health-related gas detection using 2D material-based wearable devices. **a** Schematic structural diagram of a bendable NO_2_ gas sensor based on ultrathin In_2_O_3_/g-C_3_N_4_ heterostructure. **b** Influence of light intensity on the In_2_O_3_/g-C_3_N_4_ heterostructure-based sensor performance. **c** Repeatability of the In_2_O_3_/g-C_3_N_4_ heterostructure-based sensor toward 100 ppb and 1 ppm NO_2_ at room temperature under visible light illumination. Reproduced with permission: Copyright 2021, Wiley–VCH [221]. **d** Schematic structural diagram of the PtSe_2_-based NH_3_ sensor. **e** Comparison of limits of detection (LODs) of PtSe_2_-based NH_3_ sensor in a flat state and at 1/4 mm.^−1^ curvature strain. The inset shows the 3-cycle response test under 50 ppb NH_3_. **f** Performance comparison with other transition metal dichalcogenides (TMDs)-based gas sensors. Reproduced with permission: Copyright 2023, American Chemical Society [222]. **g** Structural diagram of SnS_2_-based NO sensor. **h** The sensing performance for different concentrations of NO in SnS_2_-based sensor. Note how the sensitivity reverses from negative to positive for NO concentrations ≥ 75 ppb. **i** Sensitivity in SnS_2_-based NO sensor as a function of different exhaled NO concentrations. Reproduced with permission: Copyright 2021, AAAS [223]

Numerous researchers have made much effort to realize the rapid detection of trace amounts of harmful nitrogen dioxide (NO_2_) gas in the atmosphere [229]. For instance, Choi et al. [230] developed a wearable sensor using porous 2D Ruthenium oxide (RuO_2_) nanosheets, which exhibited a reliable and consistent electrochemical response to NO_2_, even at concentrations as low as 20 ppm at 80.3 ºC. Inspired by artificial neural networks, Chen et al. [231] constructed a wearable sensor based on n-p heterojunction of CuS quantum dots and Bi_2_S_3_ nanosheets, achieving a notably high response value with a potential limit of detection as low as 78 ppb for NO_2_. Furthermore, in pursuit of ultrafast and sensitive monitoring of NO_2_ at room temperature. Han et al. [221] prepared a flexible NO_2_ gas sensor using ultrathin In_2_O_3_/g-C_3_N_4_ heterostructure. This flexible heterostructure displayed a perfectly reversible response/recovery dynamic process, enabling the detection of NO_2_ at ultralow levels down to 50 ppb under visible-light illumination (Fig. 8a–c).

Ammonia (NH_3_) is another representative air pollutant associated with industrial production, agriculture fertilizer usage, and motor vehicle emissions. Exposure to NH_3_ concentrations exceeding 25 ppm can result in harm to the skin, eyes, and respiratory system [232]. Xing et al. [233] used graphene oxide, polyaniline (PANI), and vanadium pentoxide (V_2_O_5_) to fabricate a meticulously engineered nanocomposite textile capable of monitoring NH_3_ levels in the environment. The presence of large oxygen-containing functional groups in graphene oxide facilitates its binding with aniline monomers, thus enhancing NH_3_ sensing capabilities. The textile exhibited an augmented response, detecting NH_3_ at room temperature down to 0.5 ppm, showcasing high stability with a response retention of 91.5% after 56 days, and demonstrating excellent interference selectivity. In addition, Wang et al. [222] synthesized PtSe_2_ nanosheets in situ on a flexible polyimide (PI) substrate through thermal-assisted conversion, subsequently encapsulating them into an NH_3_ gas sensor with an ultra-low detection limit (Fig. 8b–d). This sensor displayed higher detection sensitivity (31.67% ppm^−1^) and a reduced detection limit (50 ppb) under the influence of the stress field. These enhancements primarily stem from the lattice distortion of PtSe_2_ induced by stress, which reduces the adsorption energy of NH_3_ molecules on the PtSe_2_ surface, facilitating increased charge transfer.

As the primary biomarker of asthma, monitoring nitric oxide (NO) levels serves as a fundamental method for the early diagnosis and treatment of asthma [214]. However, various physical factors such as humidity (typically exceeding > 80% relative humidity in exhaled air), temperature, and pressure fluctuations are considered confounding variables that require meticulous control or elimination to accurately analyze exhaled biomarkers. In this regard, Huang et al. [223] devised a wearable gas sensor by combining liquid metal with SnS_2_ nanosheets for remote monitoring of NO in breath. This sensor exhibited excellent skin compatibility and high responsivity, detecting ultra-low NO concentrations as low as 1.32 ppb. The excellent sensing performance can be attributed to the high adsorption energy, efficient charge transfer, and unique molecular structure variation, comprehensively facilitated by the 2D SnS_2_ layered structure. In addition, SnS_2_ nanomaterials effectively interact with water vapor in exhaled air, allowing for more precise identification of respiratory status and aiding in the prevention of respiratory distress. Due to the distinct redox properties of H_2_O and NO molecules, the sensor issues an early warning once the NO concentration exceeds 58 ppb, thereby aiding in the early diagnosis and treatment of lung diseases (Fig. 8g–i).

Volatile organic compounds (VOCs) represent an essential category of toxic gases capable of rapidly diffusing into the environment and posing severe health risks even at low (sub-ppb) concentrations. Generally, there are about 50–300 types of VOCs in indoor air environments such as homes, schools, and offices, due to the widely used interior decorations, artificial consumers, and personal care items in our daily lives [234]. Methanol, among the most common VOC, readily volatilizes from aqueous solution and even minor exposure through inhalation or skin contact can lead to headaches, blindness, and even death. Traditional methods of methanol analysis mainly rely on bulky and expensive analytical instruments [234] or detection of other physical properties of methanol/water mixture [235], such as surface tension, refractive index, density, and optical absorptivity, which hamper real-time wireless detection. Using evenly dispersed Pt nanoparticles on rGO nanosheets, Ma et al. [236] printed a wearable sensor for methanol monitoring. GO, with its excellent chemical and physical properties including structural flexibility, ultrathin thickness, and chemical stability, serves as an ideal carrier for well-dispersed Pt nanoparticles. This sensor demonstrated high selectivity, sensitivity, stability, and repeatability while maintaining good bending and stretching performance in vapors and liquids with variable temperatures or humidity levels. N, N-Dimethylformamide (DMF), another commonly used industrial solvent, can induce hepatotoxicity, embryotoxicity, and even carcinogenicity via chronic inhalation [237]. Wang et al. [238] fabricated a flexible, transparent, wearable wrist sensor device using layered polydiacetylene and MoS_2_ nanosheets. This device demonstrated linear detection of DMF vapor ranging from 0.01% to 4%, with a rapid color change from blue to red perceptible naked eye can instantly recognize the DMF.

#### Chemical Signals in Body Fluids

Flexible sensors designed for body fluids, encompassing saliva, sweat, urine, and tears, have emerged as convenient and portable alternatives to conventional sophisticated analytical instruments in the healthcare sector [254–257]. These biofluids contain rich sources of vital biomarkers such as ions (e.g., Na^+^, K^+^, and Ca^2+^), small molecules (e.g., glucose, uric acid, and lactate), and proteins (e.g., cytokine), thereby providing clinically valuable insights into the management of various metabolic diseases like diabetes, gout, etc_._ [258–260]. While blood remains the primary biofluid used in traditional medical settings, the invasive nature of finger-pricking for blood extraction limits its real-time and long-term applications. In contrast, emerging wearable devices employing noninvasive biosensing technologies have attracted considerable attention for continuously monitoring several metabolites in biofluids, eliminating the need for blood contact and thereby minimizing the risk of harm or infection [21, 261, 262]. Although urine is commonly used as a clinical sample, its suitability for autonomous and continuous monitoring is limited [263]. Tears, while containing valuable biomarkers [76] like salts, enzymes, proteins, and lipids, present challenges due to current sample collection protocols potentially causing eye irritation and influencing sensor test results [264]. Similarly, saliva contains a variety of biomarkers, including hormones, enzymes, antibodies, and antibacterial agents that can accurately reflect the body's condition [265]. Unfortunately, saliva monitoring faces hurdles due to the presence of impurities such as food particles in the oral cavity, affecting data reliability. Interstitial fluid, with analyte concentrations similar to blood [266], requires invasive methods for sampling collection, leading to discomfort and tissue irritation. In contrast, sweat offers significant advantages for wearable sensing, playing vital roles in regulating the body’s thermal balance [267] and maintaining physiological functions such as thermoregulation, moisturization, immune defense, and maintaining the balance of electrolyte and pH value [268]. Rich in physiologically relevant substances [193, 269], sweat serves as a valuable source of molecular level health information [263, 270], making it ideal for continuous and noninvasive monitoring due to its widespread distribution and easy accessibility on the body.

**Fig. 9:**
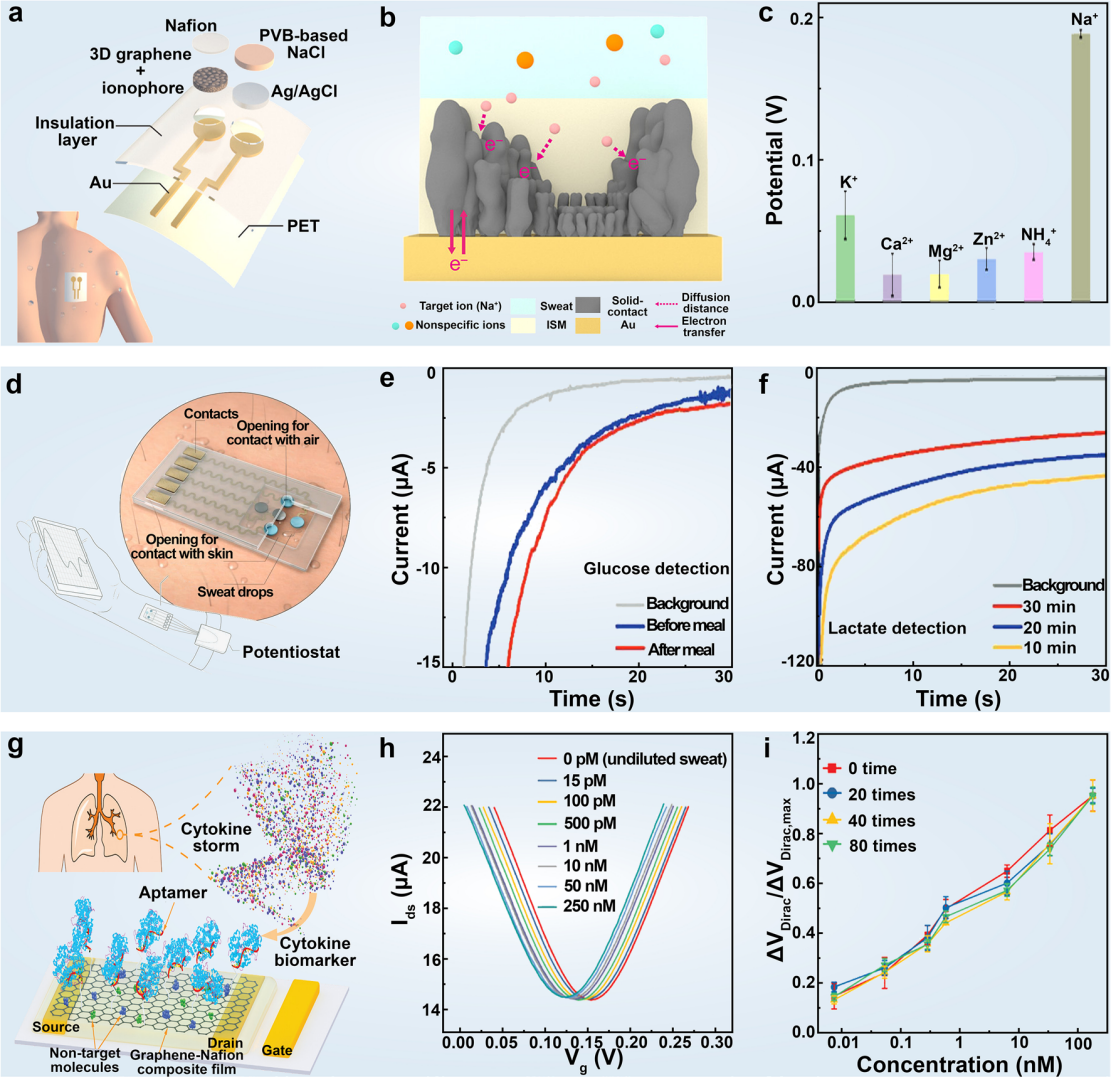
Body fluid detection with 2D material-based wearable devices. **a** Schematic diagram of the device configuration of the graphene-based sweat sensor. **b** Illustration depicting the reduction of ion diffusion paths in graphene electrodes within body fluid sensors. **c** Ion interference testing of the graphene sensor. Reproduced with permission: Copyright 2022, American Chemical Society [271]. **d** Schematic structural diagram of MXene-based body fluid sensor. **e** Measurement of glucose using the MXene-based body fluid sensor before and after meals. **f** The temporal current response of the lactate sensor at various time points during exercise. Reproduced with permission: Copyright 2019, Wiley–VCH [272]. **g** Schematic representation of the aptamer-modified graphene-Nafion field-effect transistor biosensor for cytokine biomarker detection. **h** Transfer characteristic curve measured upon exposure of the biosensor to various concentrations of IFN- γ in undiluted sweat. **i** Biosensors with different regeneration cycles were used to detect IFN- γ across concentrations ranging from 0.015 to 250 nM. Reproduced with permission: Copyright 2020, Wiley–VCH [273]

For instance, cystic fibrosis, a disease characterized by sodium ion (Na^+^) imbalance in sweat, thus can be directly diagnosed by measuring Na^+^ concentration in sweat [274]. However, conventional solid-contact ion-selective electrodes and water-layer-forming electrodes typically suffer from issues such as potential drift, chemical hysteresis, and physical delamination of the sensing device. In this regard, Yeung et al. [271] used graphene-based gradient porous electrodes to achieve real-time detection of Na^+^ in human sweat, offering superior high selectivity and sensitivity. This enhancement is attributed to the reduction of ion diffusion paths, enhanced electroactive surface area, and minimized formation of water layers on graphene electrodes (Fig. 9a–c). To further improve Na^+^ monitoring sensitivity and the repeatability of wearable devices, Park et al. [275] employed graphene ink combined with polyurethane to fabricate highly conductive Na^+^ wearable sensors with excellent mechanical properties. These sensors demonstrate excellent electrochemical performance, high sensitivity, fast response, good repeatability, and long-term stability.

Uric acid (UA), a vital biomarker in sweat, is closely associated with inflammatory responses triggered by hyperuricemia and gout. Electrochemical methods are particularly advantageous for wearable UA detection owing to the intrinsic electrochemical activity of UA and its easy oxidation tendency with minimal interference from other analytes in sweat at physiologically relevant concentrations. Although some researchers have used uricase-modified electrodes for UA detection [276], transition metal electrodes pose stability and biocompatibility concerns, and enzymes are relatively expensive and sensitive to temperature variations. Carbon-based enzyme-free electrodes offer a promising solution due to their excellent performance, cost-effectiveness, and biocompatibility [263]. Zhang et al. [277] used gold clusters and chitosan to establish a superhydrophobic and superconductive interface on graphene, enabling simultaneous monitoring of UA and pH with high selectivity, accuracy, and a low detection limit among various sweat interferents. The resulting sensor can continuously monitor sweat for at least ten days, providing insights into daily purine intake, and offering the potential for gout precautions. Accurate detection of glucose levels in body fluids is crucial for enhancing the longevity and quality of life of diabetic patients, given the myriad complications associated with insufficient insulin secretion [262]. To this end, Lei et al. [272] developed a stretchable, robust, and sensitive wearable sensor using MXene/Prussian blue (Ti_3_C_2_T_x_/PB) (Fig. 9d–f). Firstly, Ti_3_C_2_T_x_ serves as a sensitive detection interface facilitating rapid biomolecule immobilization. Secondly, Ti_3_C_2_T_x_ exhibits excellent electrical conductivity and electrochemical activity, leading to significantly enhanced detection capability compared with graphene/Prussian blue and carbon nanotubes/Prussian blue composites. Notably, the positively charged surface, catalytically active adsorption sites, excellent permeability, and tunable microenvironment of layered double hydroxides and their derivative structures make such 2D nanostructures highly effective for detecting various small molecules as conventional biomarkers, as systematically reviewed elsewhere [278].

Cytokines are critical biomarkers of trauma, sepsis, cancer, and rheumatic diseases [279]. Precise detection of trace amounts of cytokine levels in body fluids holds immense potential for distinguishing patients with worsening acute infectious diseases and monitoring overall health in daily life. Traditional cytokine detection methods, such as immunofluorescence and enzyme-linked immunosorbent assay, are hindered by their time-consuming nature, bulkiness, and complexity, making them unsuitable for integration into wearable sensors. In contrast, miniaturized detection devices like electrochemical sensors necessitate dilution of the analytes, rendering them susceptible to challenges posed by low cytokine concentrations and background interference [280, 281] in human biofluids such as sweat, tears, and saliva. Therefore, the development of wearable sensors capable of sensitively and continuously detecting cytokines in undiluted human body fluids holds profound significance for facilitating convenient monitoring of patients' conditions in daily life [279, 280]. In pursuit of this objective, Wang et al. [273] developed a flexible and repeatable aptamer field-effect transistor biosensor comprising a graphene-Nafion composite film. This innovative design effectively mitigates non-specific adsorption and confers regenerative capabilities to graphene transistor biosensors. This sensor was able to detect cytokine storm biomarkers, including interferon-γ, an inflammation and cancer biomarker, in undiluted human biological fluids with a detection limit as low as 740 f_M_ (Fig. 9g–i).

## Smart Wearable Devices

With the advancement of health monitoring technology, the development of smart wearable devices for continuous health monitoring and recording physical activity has become essential. Traditional wearable devices generally rely on external power sources to monitor human physiological signals. However, spurred by the growing demand in the healthcare device market together with the extensive research efforts on 2D materials, and new manufacturing technologies, there has been a remarkable surge in the development of various intelligent wearable devices. These include self-powered wearable devices [285–287], artificial intelligence wearable devices based on machine learning [135, 288, 289], wearable devices integrating disease diagnosis, treatment [290], and other functions [291–293]. The frontier of research lies in the smart functionalization of these devices, with their applications holding tremendous promise for enhancing human living standards and quality of life.

### Self-powered Wearable Devices

Self-powered wearable devices, capable of harnessing energy from various environmental sources such as mechanical, solar, chemical, etc*.*, via converting them into internal power, have generated significant interest in the development of next-generation portable, integrated, and miniaturized healthcare electronics [294–297]. This approach presents an insightful design principle that addresses the inconvenience of external batteries at the source, reduces the size of multifunctional integrated biological devices, and enhances sensor portability [298–300]. Wearable devices have shown excellent effectiveness in health monitoring and disease prevention, particularly as improvements in comfort, durability, and functionality have enhanced user compliance. Advances in technology have led to multifunctional, self-powered sensors that support long-term health management for chronic diseases like diabetes, asthma, and heart disease, offer preventive recommendations, and monitor medication adherence. Additionally, self-powered devices fully meet the requirements of human–machine interaction design and can be manufactured to be lightweight, structurally adaptable, and highly flexible, satisfying the needs of next-generation IoT biosensing.

**Fig. 10:**
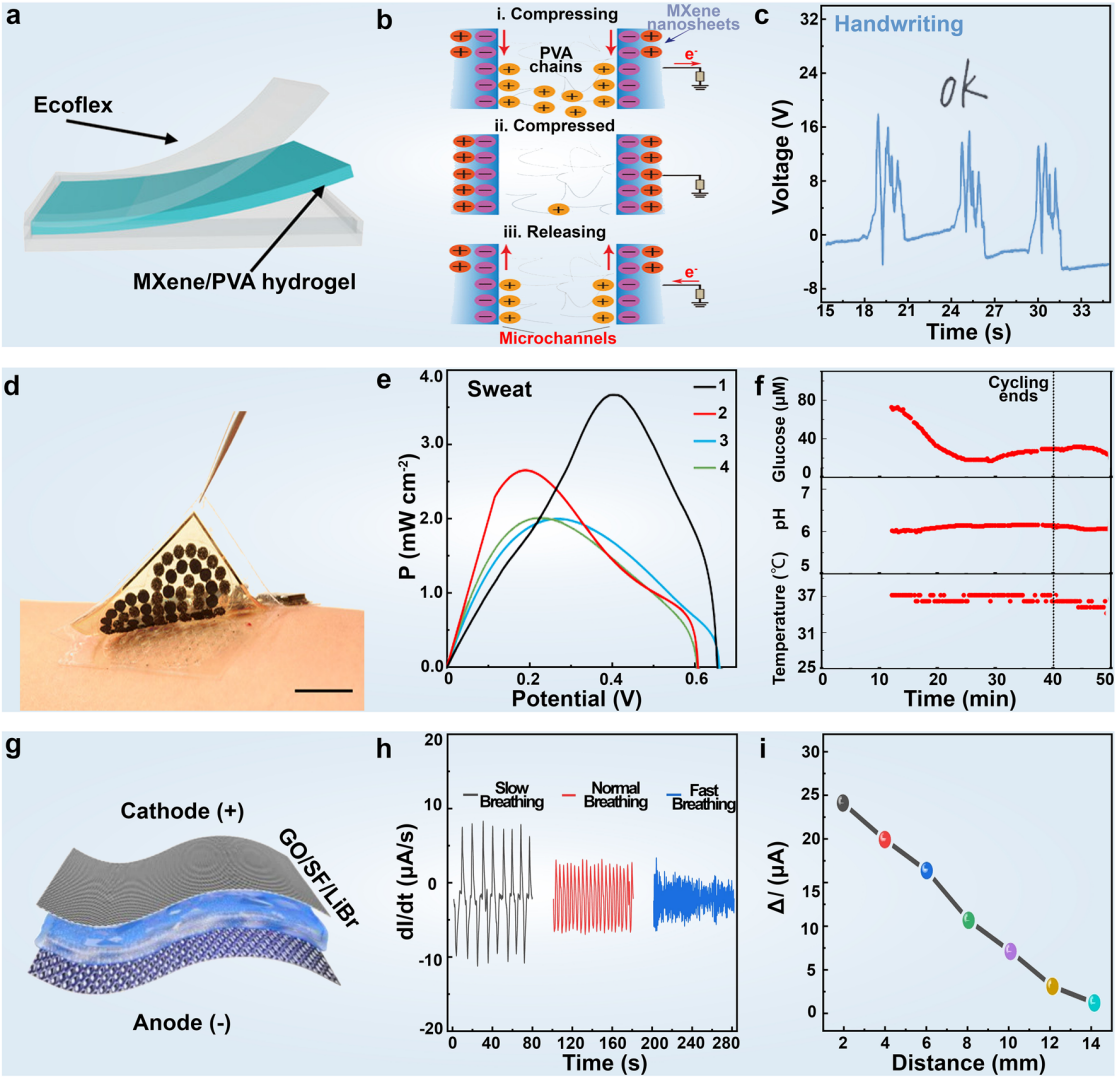
Self-powered wearable 2D material-based wearable devices. **a** Structural diagram of MXene/PVA self-powered device. **b** Triboelectric charging mechanism based on MXene/PVA hydrogel microchannels. **c** MXene/PVA-based smart wearable devices for handwriting recognition. Reproduced with permission: Copyright 2021, Wiley–VCH [282]. **d** Schematic of battery-free, biofuel-powered electronic skin. **e** Power density curves of biofuel cells in sweat samples sourced from four healthy humans. **f** Graphene-based sensors for real-time temperature, pH, and glucose monitoring. Reproduced with permission: Copyright 2020, AAAS [283]. **g** Structural diagram of GO-based self-powered sensor. h) The GO-based self-powered sensor detects respiratory signals at different frequencies. **i** Variations in short-circuit current (Δ*I*) of the GO-based self-powered sensor at different distances above the water surface. Reproduced with permission: Copyright 2022, Springer Nature [284]

Piezoelectric and triboelectric effects can convert the mechanical energy generated by the human body into electricity, making them ideal candidates for monitoring various health signals related to human movement without requiring an external power supply [301]. However, several key challenges need to be addressed, including enhancing the conformability of sensors with human tissue to minimize mechanical energy loss, achieving high sensitivity to target physiological signals amidst other disruptive body movements, and ensuring the accuracy and stability of sensors for long-term monitoring. Intriguingly, several proof-of-concept applications based on the triboelectric effect have efficiently converted biomechanical motions, like expiration airflow, hand movements, walking, and joint flexion, into electrical signals for bio-energy harvesting and health-signal detection. These miniaturized devices hold potential for integration with mobile terminals to enable the monitoring of various physiological parameters [302]. Utilizing the excellent electrical characteristics of 2D materials, Chen et al. [303] used borene/ecoflex nanocomposite material to construct fabric-based smart wearable devices with self-power supply capabilities. The borophene/ecoflex nanocomposite offers advantages such as high surface charge density, cost-effectiveness, scalability, flexibility, and durability. Borophene nanosheets enable tunable surface triboelectricity within the nanocomposite layer, as demonstrated by Kelvin probe force microscopy. This smart wearable sensor, integrated with a robotic system, was employed for upper-limb medical assistive interfaces and active gait phase sensing system, allowing for immediate visualization of lower-limb gait phases. Notably, the smart wearable can simultaneously function as a self-powered external electrical stimulation device for continuous wound monitoring and therapy. In addition, Luo et al. [282] created a hydrogel smart wearable device with facile fabrication and excellent output performance by incorporating MXene and PVA. The dispersion of MXene nanosheets within the hydrogels promoted crosslinking and stretchability, while also enhancing ion transport and conductivity through the formation of microchannels on the surfaces. This facilitated triboelectric output via the streaming vibrational potential process. These self-powered integrated devices revealed versatile applications in wearable motion monitors and highly accurate stroke recognition (Fig. 10a–c). Building upon this foundation, Long et al. [304] introduced polyacrylamide into MXene and PVA system to prepare a hydrogel intelligent wearable device with ultra-high mechanical strength, super elasticity, and excellent fatigue resistance. The hydrogen bonding between Ti_3_C_2_T_x_ nanosheets and polymer chains played a crucial role in achieving these unique physical properties. Notably, the introduction of Ti_3_C_2_T_x_ nanosheets not only enhanced the electrical conductivity of hydrogel but also facilitated the formation of an electrical double layer (DEL) between the MXene nanosheets and the free water molecules within the hydrogel. When the hydrogel is under external pressure, a potential difference is generated between the two surfaces of the electrified layer, creating an induced electric field between the two sides of the hydrogel. This allows current to flow through copper wires from the external circuit into the hydrogel, enabling the output of triboelectricity. This enhancement in electric output generation via DEL contributed to the utilization of device for human motion monitoring, including gesture recognition, finger bending, vocal cord vibration, and facial expression analysis.

With the growing popularity of outdoor sports, there is a pressing need for intelligent wearable devices capable of high sensing sensitivity and stable operation in challenging conditions, particularly for wilderness exploration and emergency rescue scenarios. Zhao et al. [305] developed an anti-freezing, stretch-matchable, and liquid electrode-based triboelectric nanogenerator tailored for biomechanical sensing in frigid environments. This device features a single electrode fabricated by integrating lithium chloride electrolyte, graphene oxide micro-/nano-sheets, and ethylene glycol, enabling a high stretchability of up to 200% and high electrical performance output. In the liquid electrode, GO sheets as a kind of electronic conductor have abundant functional groups, and thus can be dispersed well in water and ethylene glycol to improve the conductivity. The sensor reveals two distinct advantages: it functions as a power source for self-powered wearable sensors, monitoring biomechanical motion when attached to the human skin, and maintains robust performance in harsh environments across a wide temperature range ( -40 to 25 °C) without compromising its sensing capability [86].

While wearable devices integrating self-powered modules have demonstrated diverse functionalities, including multi-channel sensing, on-demand driving, and wireless data transmission over prolonged periods, these encounter significant energy consumption challenges [306]. This is largely due to the limitations inherent in self-powered wearable devices based on mechanical and thermal energy, such as low power density and discontinuity in energy collection and conversion processes [307, 308]. In this context, Yu et al. [283] reported a flexible and fully perspiration-powered integrated electronic skin (PPES) for the in-situ sensing of multiplexed metabolism. This battery-free electronic skin featured multimodal sensors and highly efficient lactate biofuel cells, incorporating a distinctive integration of nanomaterials to achieve both high power intensity and long-term stability. It delivered a record-high power density of 3.5 mW cm^−2^ for a biofuel cell operating in untreated human body fluid (human sweat), showcasing remarkably stable performance over a continuous operation of 60 h. Furthermore, it selectively monitored key metabolic analytes (e.g., urea, NH_4_^+^, glucose, and pH) and skin temperature during long-term physical activities, seamlessly transmitting the data to the user interface via Bluetooth. Additionally, the PPES served as a dual-function human–machine interface, monitoring muscle contraction and facilitating human prosthesis walking (Fig. 10d–f). Notably, in addition to being a physiological signal for healthcare monitoring, human body temperature generates a continuous heat energy output of 40 mW cm^−2^ [309], making thermoelectric materials compelling candidates for body heat energy harvesting and providing a sustainable source for wearable bioelectric devices. The intriguing anisotropic properties in 2D materials such as MoS_2_, WS_2_, MXene, SnSe, and others [310], especially in their nanocomposites, are widely studied through both experimental demonstrations and theoretical simulations to enhance thermoelectric performance [311].

In addition to harnessing mechanical energy from human motion, body heat [309] and biological energy from perspiration for self-powered wearable technology [312], chemical energy presents another viable option. Inspired by the metal-air redox reaction commonly utilized in metal-air batteries, Li et al. [284] designed a self-powered thermoelectric humidity sensor. This sensor features a silk fibroin (SF) and LiBr gel matrix infused with parallelly aligned graphene oxide (GO) flakes, serving as the electrolyte. The presence of large hydrophilic groups in GO/SF, coupled with the hygroscopic nature of LiBr, results in a strong correlation between the output current and humidity levels, facilitating high sensitivity, rapid response, and swift recovery of the sensor. As a proof of concept, the study demonstrated an all-in-one respiratory monitoring-diagnosing-treatment system and a noncontact human–machine interface, showcasing the versatility and potential applications of the thermoelectric humidity sensor in health management **(**Fig. 10g–i).

### Human–Machine Interaction

Human–machine interaction (HMI) technology has attracted extensive attention for its pivotal role in applications in the Internet of Things (IoT), such as wearable electronics [21, 313] and remote medical monitoring [193]. Intelligent sensors serve as linchpins in HMI systems, adeptly translating various signals from the human body into machine-readable information [314]. Therefore, the development of various HMI sensors with high sensitivity and rapid response is essential. On one hand, HMI act as communication pathways between humans and machines, capturing physical or physiological electrical signals from the user and driving the system to perform specific functions. Traditional control terminals include keyboards, touchpads, and joysticks, while more diverse wearable devices meet additional demands of technological advancement, such as smart prosthetics, automatically adjusting glasses, and interactive smart gloves. Many intriguing proofs-of-concept, such as eye-control devices [315], have been extensively reported, providing promising prototypes for integrating more innovative functionalities into wearable devices based on 2D materials [316]. On the other hand, the application of HMI technology in intelligent recognition has received widespread attention due to its high sensitivity and fast response in voice, facial, and integrated biometric recognition, offering new possibilities for secure identification. Utilizing the unique properties of 2D materials, particularly their heightened sensing sensitivity, is crucial in the advancement of wearable biodevices for HMI applications [288, 317].

**Fig. 11:**
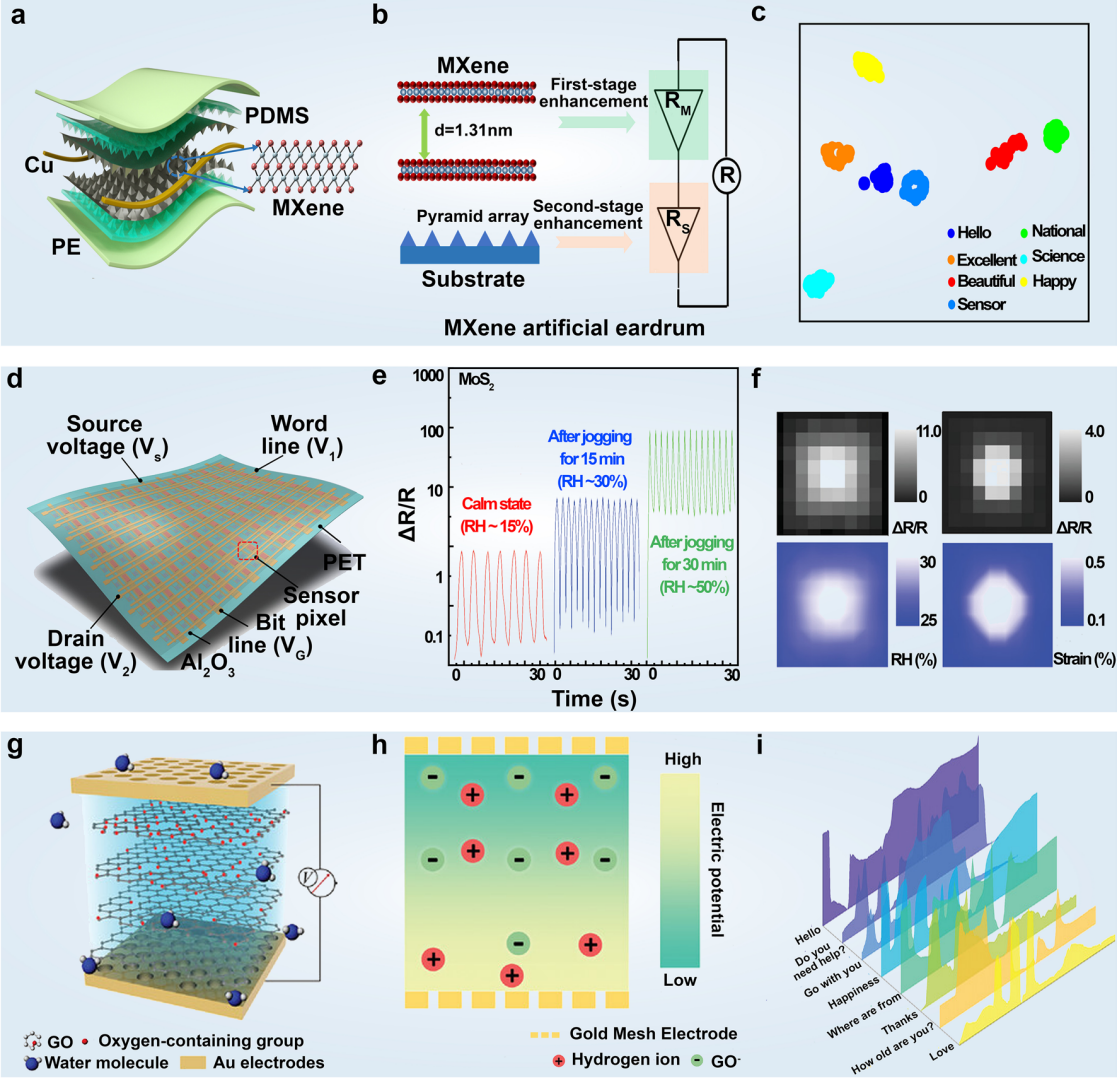
Human–machine interaction with 2D material-based wearable devices. **a** Schematic diagram illustrating the three-dimensional structure of the cochlear implant based on MXene. **b** The operating principle of two-stage enhancement in MXene-based HMI devices. **c** Visualization of the pronunciation information of voice within 280 voices adopting t-distributed stochastic neighbor embedding (t-SNE) dimensionality reduction. Reproduced with permission: Copyright 2022, AAAS [318]. **d** Schematic diagram of the structure of a 10 × 10 sensor array based on MoS_2_. **e** Observation of MoS_2_ resistance change rate reflecting human respiratory humidity under different exercise states. **f** Black and white plots (top) of MoS_2_ (left) and nanographene (right) resistance change rates upon finger contact with the electronic skin, while the bottom images represent the corresponding relative humidity and strain distributions, respectively. Reproduced with permission: Copyright 2021, Wiley–VCH [322]. **g** Structural diagram of GO-based HMI sensor. **h** Spontaneous movement of hydrogen ions to the bottom under gradient diffusion, generating a potential difference at both ends of the GO-based HMI sensor. **i** Sequence of pressure changes received by the sensor under eight common sign language movements. Reproduced with permission: Copyright 2022, Wiley–VCH [323]

Artificial eardrums capable of real-time speech recognition in response to tiny mechanical signals hold great promise for integration into wearable acoustic healthcare devices. Guo et al. [318] used Ti_3_C_2_T_x_ MXene to prepare an artificial eardrum with extremely high sensitivity and a detection limit as low as 0.1 Pa. Notably, a real-time audio categorization system may be realized with great accuracy owing to the ultrasensitive MXene eardrum. Machine learning methods are used to analyze MXene eardrum data recorded in various complex environments. The speech recognition algorithm based on the k-means clustering algorithm has achieved good clustering results, local optimization, and reduced complexity in data processing. Successfully classifying 280 voice signals into seven categories, this system attained a high accuracy of 96.4 and 95% for the training and test datasets, respectively (Fig. 11a–c). In addition, many metallic 2D monolayers, including transition-metal dichalcogenides and MXenes [319], have demonstrated superior electrical conductivity comparable with traditional metal electrodes. Their large surface and dangling bond-free interfacial contact enable intimate interactions with biological molecules and tissues. Nanoscale engineering of these metallic 2D materials allows for fine-tuning miniaturized biodevices with enhanced performance and integrated multifunctionalities. For instance, metallic 2D platinum diselenide and platinum ditelluride exhibit broad detectability for various human physiological vital signs, including the electrical activity of heart and brain, muscle contractions, eye movements, and temperature variation. Notably, their measured performance surmounts that of state-of-the-art gold and graphene electronic tattoos. This remarkable performance could further enhance advanced human–machine interface applications [320].

Wearable strain sensors, sensitive to joint and muscle strain, have become prevalent in human–machine interfaces for comprehensive full-body motion monitoring. However, most wearable technology lacks customization, hindering the optimization of sensor properties tailored to specific joint or muscle deformation ranges, thus resulting in suboptimal performance. Yang et al. [321] designed wrinkle-like topographies on piezoresistive MXene nanosheets. By integrating human–computer interaction and machine learning, real-time monitoring of body movement states can be accurately achieved, and displayed through animation, offering promising prospects in high-precision motion detection, athlete performance analysis, and augmented/virtual reality applications.

Artificial electronic skin possesses excellent application prospects in physical therapy and human–computer interaction, yet challenges persist in achieving high-density integration, ultra-sensitivity, and multi-functionality. To this end, Zhao et al. [322] reported a multimodal and comfortable skin-inspired active-matrix circuitry, utilizing all 2D MoS_2_ materials with high pixel density (> 100 cm^−2^). This sensor array exhibits excellent performance in detecting both mechanical interactions and humidity variations, with ultra-high sensitivity (gauge factor > 400 for strain and ≈10^4^ for humidity sensing), long-term stability (> 1000 cycles), and simultaneous multi-stimulus sensing capability with rapid response time. Accordingly, a respiratory monitor constructed based on this device enables real-time healthcare monitoring by observing human breath frequency, intensity, and humidity. Moreover, the multimodal e-skin breaks through the shackles of the contact sensor mediums for HMI, allowing for intricate simultaneous multimodal monitoring critical for envisioning and developing a future human–machine fusion world (Fig. 11d–f). However, integrated multi-sensor networks often suffer from cumbersome structures, high power consumption, and complex preparation processes, limiting their practical applications. Yang et al. [323] tackled this challenge by developing a graphene oxide single-component multimodal sensor capable of simultaneously monitoring multiple environmental stimuli with a unique moist-electric self-power supply. This sensor can generate a sustainable moist-electric potential by spontaneously adsorbing water molecules from the air, responding similarly to different stimuli. Taking advantage of a well-designed machine learning model, the single monitoring signal representing mixed responses can be decoupled, enabling simultaneously monitoring temperature variations (20 ~ 60 °C), relative humidity (60% ~ 90%), pressure (0 ~ 2 N), and light intensity (0 ~ 200 mW cm^−2^), respectively (Fig. 11g–i).

To achieve three-dimensional (3D) interaction and multi-scene HMI, Xu et al. [324] used cellular graphene to prepare a smart wearable device capable of monitoring electrooculogram and tactile perception. By applying machine learning techniques, this device revealed an impressive 92.6% accuracy in predicting nine different eye movements. Furthermore, an ultrathin (90 μm), stretchable (~ 1000%), and flexible tactile sensing interface, comprised of a pair of 4 × 4 planar electrode arrays attached to the arm, facilitated 2D motion control and Z-axis interaction, enabling functionalities such as single-point, multi-point, and swipe touch capabilities. Notably, this flexible and ultra-thin tactile sensor exhibited an exceptional sensitivity of 1.428 kPa^−1^ within the pressure range of 0–300 Pa, alongside long-term response stability and repeatability.

### Integration of Diagnosis and Treatment

**Fig. 12:**
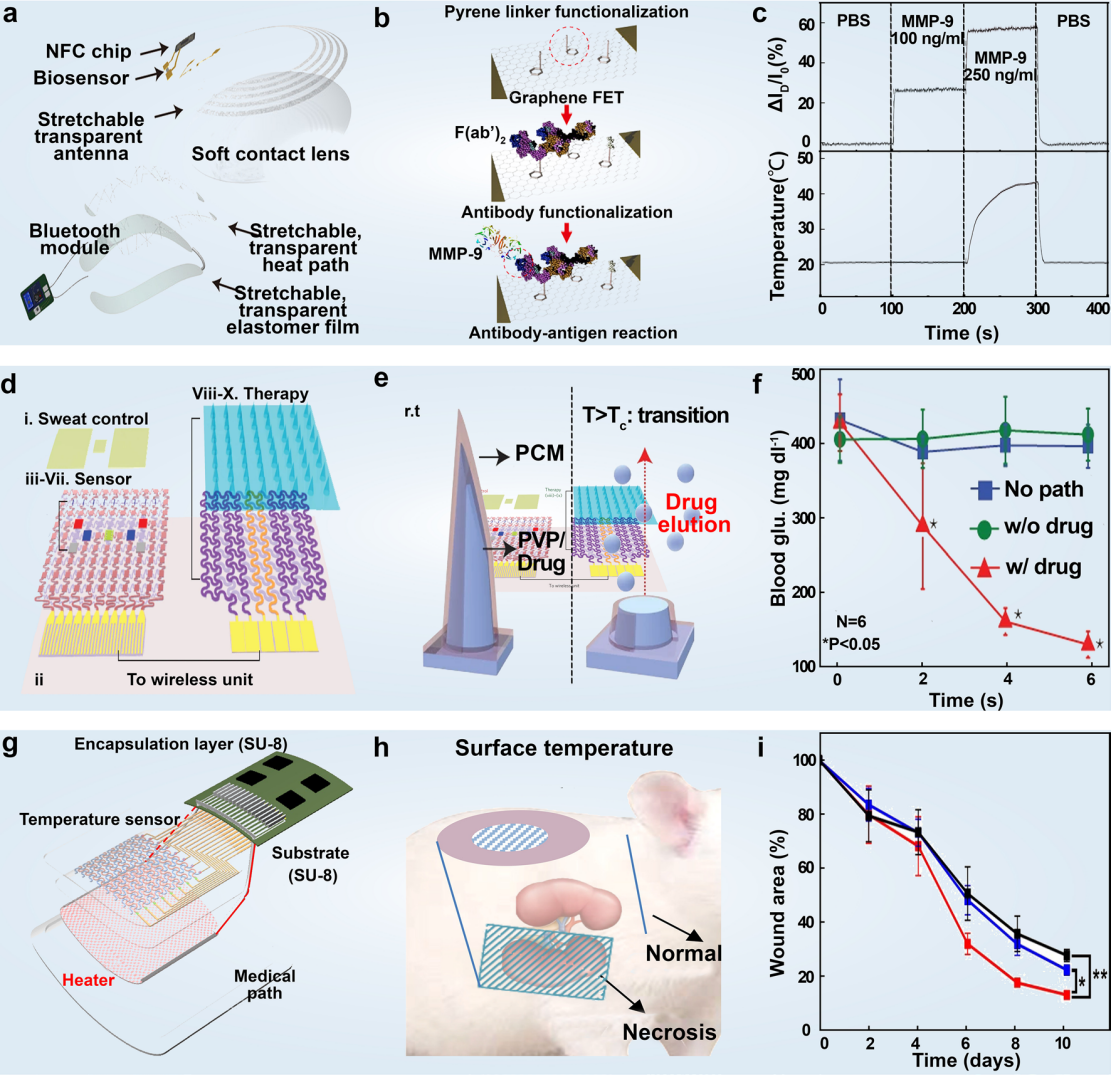
Integrated diagnosis and treatment devices using 2D materials. **a** Schematic diagram of an integrated system comprising diagnostic and therapeutic devices for real-time monitoring and treatment of chronic OSI. **b** Schematic diagram of Fab functionalization and antigen–antibody reaction of a graphene FET. **c** Detection of MMP-9 concentration and the instantaneous temperature control of the heat patch. Each plot shows real-time changes in the relative drain current of the contact lens (top) and the temperature of the heat patch (bottom). Reproduced with permission: Copyright 2021, AAAS [330]. **d** Schematic drawings of the diabetes patch, including the sweat-control (i, ii), sensing (iii–vii), and therapy (viii–x) components. **e** Schematic illustrations of bioresorbable microneedles. **f** Blood glucose concentrations of diabetic mice for the treated group (with the drug) and control groups (without the drug or patch). Reproduced with permission: Copyright 2016, Springer Nature [331]. **g** Structural schematic diagram of diagnosis and treatment system based on graphene. **h** Experiment setup for the diagnosis of kidney necrosis. **i** Comparison of average wound area over 10 days in three different groups (without patch, with patch but not heated, and with patch and heated group). Reproduced with permission: Copyright 2022, AAAS [332]

Disease treatment is equally essential as a diagnosis because timely interventions are imperative for improving patients' quality of life and survival rates. Integrating diagnosis and treatment modules within wearable devices presents the future trajectory of healthcare technology. Notably, wearable devices based on 2D materials offer unique advantages in realizing these dual functions. Their ultrahigh response sensitivity makes them particularly well-suited for precise monitoring and diagnosis [325, 326]. Moreover, the exceptional thermal conductivity, photothermal conversion efficiency, and massive surface area endow 2D materials with great promise in thermotherapy and drug delivery applications [327]. For instance, the concentration of metalloproteinase-9 (MMP-9) in tears is a crucial indicator of chronic ophthalmia, underscoring the importance of its early diagnosis for early treatment [328, 329]. In this regard, a soft, smart contact lens and a skin-attachable therapeutic device were developed for wireless monitoring and treatment of chronic ocular surface inflammation (OSI) in human trials [330]. The smart contact lens allows for real-time tracking of MMP-9 concentration in tears using a graphene field-effect transistor (FET) for diagnosis. Additionally, a flexible, transparent heat patch, comfortably attached to the eyelid, serves as a therapeutic device. Smartphone integration enables wireless coordination between diagnostic and therapeutic modalities, facilitating rapid OSI diagnosis and automated hyperthermia treatments (Fig. 12a–c).

Monitoring glucose concentration in the sweat of diabetic patients and providing timely treatment is pivotal for improving their quality of life and overall health. Lee et al. [331] fabricated wearable patches using gold-doped graphene and gold mesh, facilitating sweat-based diabetes monitoring and feedback therapy. Beyond the fundamental sensor capabilities encompassing temperature, humidity, glucose, and pH monitoring, the patches featured polymeric microneedles capable of transcutaneous drug delivery upon thermal activation **(**Fig. 12d–f).

Effective temperature monitoring and control during hyperthermia are critical for ensuring human comfort and safety. While traditional thermotherapy pads mainly focus on orthopedic and muscular concerns beneath the skin, research addressing epidermal trauma and bacterial infectious diseases is urgently required for advancing mobile healthcare and personalized medication and therapy. By employing MXene as the active material, Zhao et al. [333] converted commercial cellulose fiber nonwoven into multifunctional MXene-based smart fabrics (M-fabric). These fabrics exhibit sensitive and reversible humidity responses, facilitated by channel expansion/contraction induced by ·OH between MXene interlayers, thus enabling wearable respiration monitoring. In addition, owing to its rapid and consistent electrothermal response, the M-fabric serves as a low-voltage hyperthermia platform, effectively transferring generated heat to bacterially infected skin, thereby accelerating the healing of infected wounds. In a separate study, Gong et al. [334] used MXene nanosheets, polydopamine/Ni^2+^, and spandex yarns to fabricate wearable and sewable smart preventive textiles. This textile exhibited high sensitivity, a wide sensing range, and a low limit of detection for strain. It integrated human movement tracking, photothermal therapy, and antimicrobial activity into a single device. Thermography provides valuable insights into the body's general health condition and aids in diagnosing various ailments. Kang et al. [332] reported a wearable sensor with dual functionalities: continuous skin temperature sensing and thermal therapy. This sensor array can continuously monitor temperature variations across large skin areas during physical activity, providing high-resolution and sensitivity in health issue detection. Additionally, the incorporation of a graphene heater beneath the temperature sensor accelerates the healing process of injured skin, potentially via vasodilatory effects (Fig. 12g–h).

## Others

Wearable devices with skin-like mechanical properties have revolutionized continuous monitoring of the human body. However, prevalent wearable device designs have mainly focused on capturing surface signals from the skin, offering only a limited glimpse into health and disease. Deep tissue signals, such as electrophysiological, metabolic, circulatory, thermal, and mechanical parameters, often show stronger correlations with disease progression and symptom onset. Yet, accessing such signals remains challenging due to the strong shielding effect of the integument and musculoskeletal system. In this context, researchers have developed numerous implantable, wearable devices hinging on two-dimensional materials to probe deep tissue within the human body, thereby promoting the development of wearable devices from external to internal monitoring paradigms.

**Fig. 13:**
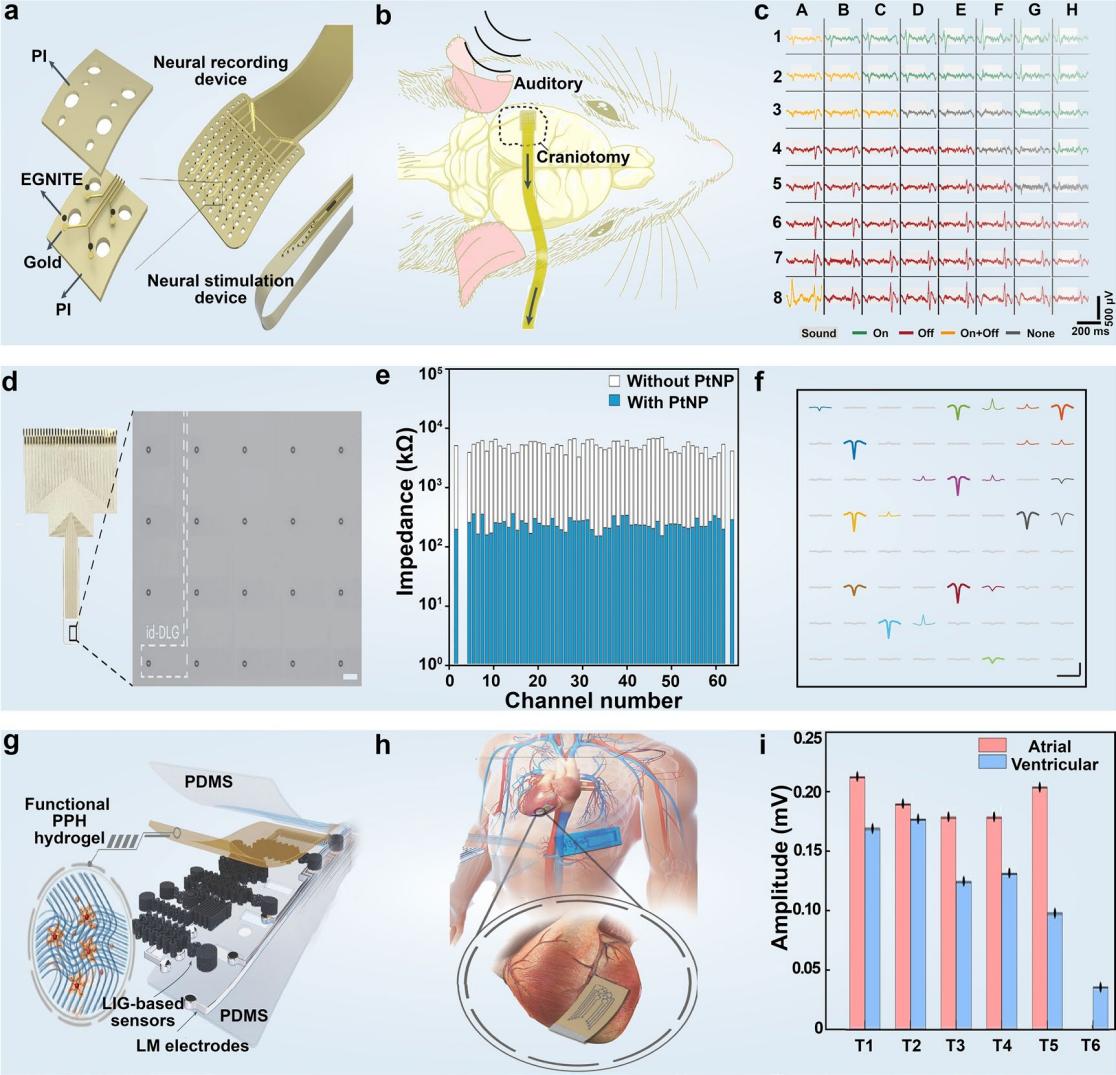
Implantable wearable devices based on 2D materials. **a** Schematic diagram of the structure and multi-array design of the graphene-based neural interface. **b** Schematic representation of an acute experiment using engineered graphene for neural interface micro-electrocorticography flexible array to record epicortical neural activity in a rat. The evoked activity was induced by pure tone stimuli. **c** Plots showcasing evoked neural activity in response to 16 kHz stimulation, depicting individual events on all 64 microelectrodes. Recorded responses include onset (green), offset (red), and both (yellow) or no onset/offset (dark gray) responses are recorded. Reproduced with permission: Copyright 2024, Springer Nature [345]. **d** Transparent and flexible 64-channel graphene array, with an enlarged section displaying graphene lines shown as white dashed lines. Scale bar, 100 μm. **e** Impedance distribution of 64 channels at 1 kHz measured before and after the deposition of platinum nanoparticles (PtNP). The average impedance of the electrodes before and after PtNP deposition was 5.4 ± 1.1 and 250 ± 56 kΩ (mean ± standard deviation), respectively. **f** Representative events on different channels trigger multiunit activity waveforms. Scale bar, 2 ms (horizontal) and 20 μV (vertical). Notably, multiple nearby channels also capture neural events, and they are color-coded to correspond with the target channel. Reproduced with permission: Copyright 2024, Springer Nature [346]. **g** Structural diagram of the laser-induced graphene heart patch. **h** Schematic representation of the cardiac patch utilized for signal monitoring such as arrhythmia. **i** Average amplitude of the atrial and ventricular signals recorded during 10 min in moribund rats. Reproduced with permission: Copyright 2024, Springer Nature [347]

Neural interface medical devices provide treatment options for patients with certain neurological diseases and nerve injuries, such as Parkinson's disease [335], deafness [336], or amputation [337]. Many studies advocate for the miniaturization of electrode size to the micron level [338, 339], facilitating the capture of neural signals with higher spatial resolution, thereby potentially bolstering neural signal decoding capability [340–342]. In addition, shrinking electrode dimensions have proven viable in improving the stimulation focus and reproducing the natural activation pattern of healthy nerve tissue. However, prevailing clinical technologies predominantly rely on utilizing millimeter-scale metal electrodes for recording or stimulating the nervous system, thus severely impeding the progress of neural interface medical devices. Graphene-related materials have become attractive candidate materials for bidirectional neural interface electrodes owing to their unique performance attributes [338, 343, 344]. Despite graphene microelectrodes have been employed in neural interface applications, their limited electrochemical performance constrains their suitability for miniaturization endeavors [343]. In this regard, Viana et al. [345] prepared a flexible neural interface based on nano-porous graphene. This engineered graphene for neural interface (EGNITE) involves microelectrodes as diminutive as 25 μm in diameter, with a low impedance of 25 kΩ and a high charge injection performance of 3–5 mC cm^−2^. In addition, it shows commendable fidelity in brain recording, low current thresholds, high selectivity in nerve stimulation, and excellent biocompatibility (Fig. 13a–c). Optically transparent nerve microelectrodes facilitate the simultaneous recording of brain surface electrophysiology with optical imaging and neural activity stimulation. Ramezani et al. [346] developed a transparent graphene microelectrode with an ultra-small opening, a large transparent recording area, and a high-density microelectrode array with up to 256 channels. Overcoming the quantum capacitance limitation of graphene entailed using platinum nanoparticles, reducing microelectrode diameter to 20 μm, and introducing interlayer doped double graphene to avert open circuit failure. The compact size and expansive coverage of graphene electrodes enable the detection of low-frequency and high-frequency activity on the cortical surface with high temporal and spatial resolution, thereby elucidating their correlation with deep-cell calcium activity (Fig. 13d–f).

Lu et al. [347] reported a thin, elastic conductive nanocomposite formed by laser-induced graphene transfer onto hydrogel films at low temperatures. The conductive environment at low temperatures enhances interfacial bonding between the defective porous graphene and the crystalline water within the hydrogel. Using hydrogel as both an energy dissipation interface and an out-of-plane electrical path, continuous deflection cracks are induced in laser-induced graphene, leading to a more than fivefold increase in its inherent tensile properties. This method yields a multifunctional wearable sensor for skin monitoring and a heart patch for in vivo detection. The heart patch seamlessly adheres to the rat heart, enabling the diagnosis of arrhythmias and identification of signal evolution in the terminal state (Fig. 13g–i).

## Conclusions and Outlook

**Fig. 14:**
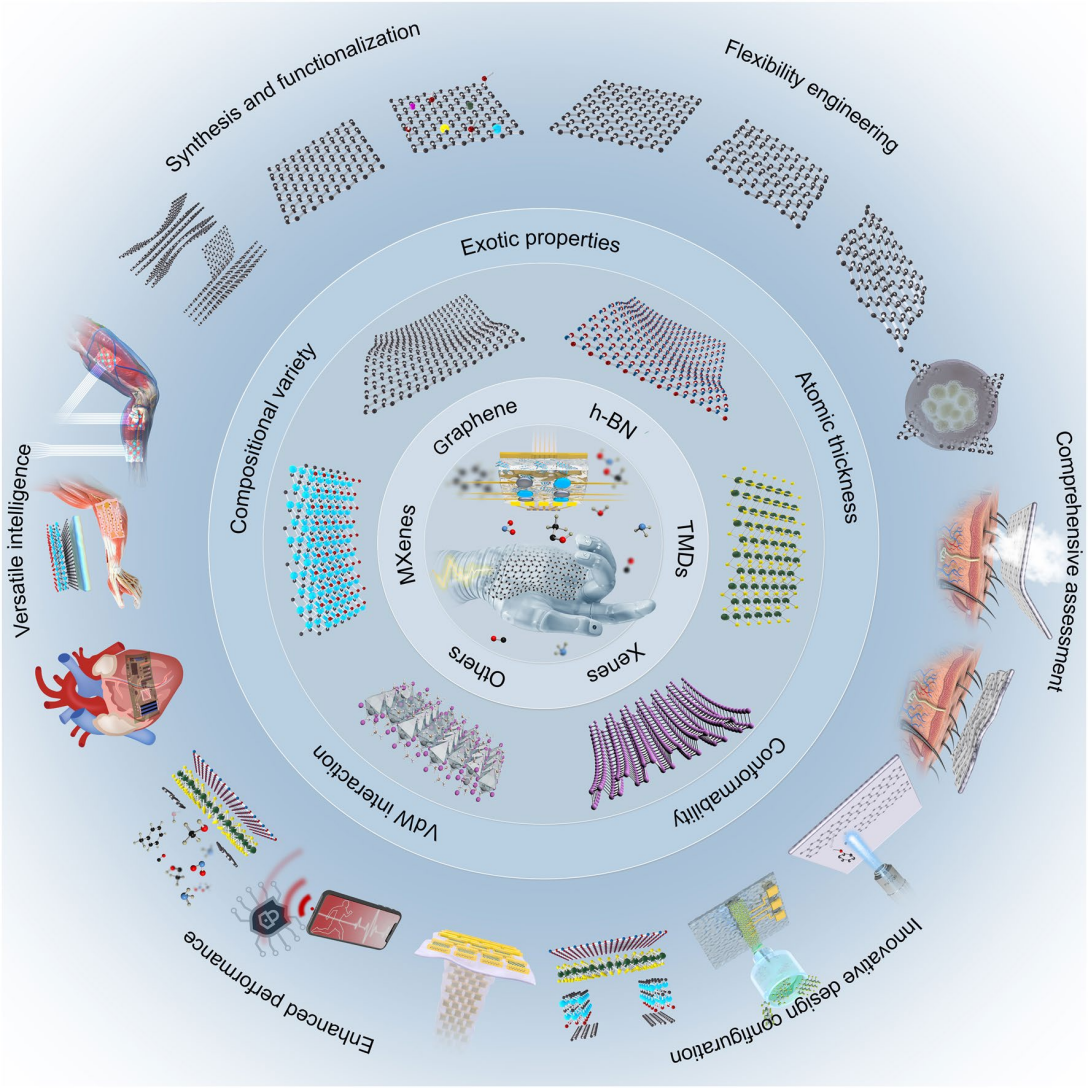
Strategies for constructing high-performance wearable devices with 2D materials

Overall, the strides made in 2D material manufacturing techniques [348, 349], surface engineering [350–352], device design [5, 40], and multifunctional integration [73] have significantly propelled the advancement of flexible wearable devices. The unique combination of exceptional mechanical flexibility and electrical properties found in atomic-thin 2D materials has endowed wearable biodevices with remarkable versatilities [353], enabling high sensitivity, low detection limit, and simultaneous recognition of various physiological signals. Moreover, the extensive 2D material library and the continuous emergence of new fabrication and integration technologies [39] have paved the way for numerous innovative applications in intelligent wearable devices. In this context, we have summarized the latest advancements in wearable biodevices based on 2D materials, spanning from flexible sensor units for human health monitoring to smart integrated devices facilitating Human–Machine Interaction, disease diagnosis, and treatment [354]. However, despite these advancements, significant challenges persist alongside abundant opportunities within this burgeoning research frontier (Fig. 14).

The broad application of bioelectronics based on 2D materials continues to face significant challenges, suggesting that the path toward integration in sensors will be long and complex. One major barrier is the scalable production of high-quality nanosheets, such as graphene, BP, and TMDs, typically produced by CVD. Additionally, the instability of 2D materials, particularly their susceptibility to oxidation and structural changes when exposed to acids, alkalis, oxygen, and moisture, raises concerns about their durability in sensor applications, often necessitating protective encapsulation. This environmental sensitivity complicates the consistency and uniformity of the sensors, thereby hindering large-scale production. Moreover, biocompatibility evaluations such as cell toxicity testing for 2D material-based biosensors are frequently overlooked. The presence of trace impurities often fails to meet the strict standards required for the transition of nanomaterials into reliable biosensors. Another challenge is the compatibility of 2D materials with soft substrates in wearable sensors, which can lead to signal interference and compromise signal stability. Furthermore, a limited understanding of the sensing mechanisms in 2D material-based biodevices restricts the development and optimization of these sensors for practical applications. In the case of graphene-based biodevices, the absence of a bandgap in graphene increases noise levels, reducing the signal-to-noise ratio in sensing applications. Additionally, the atomic thickness of graphene limits the surface area available for biomolecule attachment in implantable sensors, thereby weakening their binding strength and sensitivity. Electron transfer plays a dominant role in triboelectrification and self-powered wearable devices [309]. However, the critical roles of work function, interfacial potential barrier, and separation distance in heterostructures require systematic investigation. This requires experimental advancements to precisely control 2D material structures, like tuning surface functional groups in MXenes to determine their work functions, along with theoretical insights to address this fundamental question quantitatively.

Addressing these issues requires interdisciplinary innovations in the synthesis of 2D materials, surface functionalization, device design, assembly, and manufacturing. These advancements are critical to improving the performance and stability of 2D material-based biosensors and facilitating their widespread use.

### Controllable Synthesis and Functionalization of 2D Materials

The intricate atomic configurations of 2D materials [42, 61], encompassing factors such as nanosheet thickness, defect density, doping state, heterostructure interface, domain size/boundary, and phase transition, profoundly shape their physicochemical properties and consequently dictate the performance of assembled devices. The development of manufacturing technologies for high-quality, low-cost, and large-scale 2D materials is a crucial aspect of advancing bio-flexible sensors. However, there are currently no effective strategies to control the size and thickness of 2D materials synthesized through hydrothermal synthesis and mechanical exfoliation. Mainstream preparation strategies for large-area 2D materials include CVD, physical vapor deposition (PVD), and molecular beam epitaxy (MBE). The production of 2D materials via vapor deposition inevitably requires substrate support materials, and impurities are introduced during the transfer process, leading to performance degradation due to environmental humidity. Promisingly, efforts have focused on enhancing quality while balance scalable fabrication in the wet exfoliation process. For instance, while liquid-phase exfoliation of 2D materials has long been considered a scalable and cost-effective method for producing nanosheets with good solution processability, the limited control over structural defects in as-synthesized 2D flakes remains a significant barrier for their application in high-performance devices [355]. Recently, electrochemical exfoliation has emerged as a promising alternative [356, 357], offering a rational approach to synthesizing large quantities of 2D materials with diverse properties [358], including semiconducting [359], metallic [319], and superconducting characteristics [97]. This method presents a feasible approach to revisit traditional 2D materials and prototype device configurations, enabling a more thorough evaluation of their application potential, particularly in high-quality or intrinsic 2D materials with outstanding properties.

Achieving precise control over the structure of nanosheets and their subsequent processability is now more crucial than ever in 2D nanomaterials, ensuring both reliable measurement and enhanced sensing capabilities for complex physiological signals. However, since the isolation of graphene, a persistent dilemma has emerged concerning the delicate balance between the processability of 2D materials and the preservation of their overall properties [325, 360, 361]. Numerous chemical synthesis strategies and post-functionalization approaches have been developed to address this challenge. These encompass various techniques such as the introduction of surface oxidational functional groups [362], the utilization of dispersing solvents with tailored surface energy [363], the incorporation of surfactants to mitigate self-agglomeration [364], and the application of surface capping layers to retard oxidation [365], among others. However, while these methods have yielded advancements in enhancing the processability of 2D materials for functional device fabrication, they often come at the expense of compromising certain exotic attributes intrinsic to these materials. Despite these trade-offs, the exploration of novel approaches presents vast opportunities for further advancement [97, 366]. Additionally, the hybridization of nanomaterials often leads to new mechanical, catalytic, and electrical properties. For example, an unconventional superconductivity has been observed in bilayer graphene when the carbon atoms between the two layers are aligned at a 1.1-degree twist, resulting in a correlated insulating state with half-filling and zero resistance at a critical temperature of 1.7 K [367, 368]. This presents a new avenue for utilizing hybrid nanosheets, either of the same or different types, to create heterostructures with special properties. Therefore, advanced manipulation of the precise spatial positioning of nanomaterials is vital for triggering significantly improved performance in various applications. With the rapid development of advanced surface modification methods, the universal synthesis of such hybrid materials may become a reality, providing hybrid 2D nanomaterials with exciting properties for a wide range of applications, including biosensing. By integrating a diverse array of mechanical, electronic, photonic, magnetic, and other properties into unified platforms, the realization of multifunctional and synergistic performance in highly compact devices becomes achievable. This pursuit signifies a pivotal pathway toward unlocking the full potential of 2D materials in advanced wearable devices.

### Flexibility Engineer in 2D Material-Based Devices

Flexibility represents a fundamental requirement in wearable devices [369], serving to mitigate strain induced by body motion and ensuring seamless conformity to irregular surfaces. However, an exemplary wearable sensor should be capable of conformally monitoring bio-signals across a wide range of strains, accommodating the diverse range of body movements and activities encountered in daily activities [370]. In this regard, customized flexibility in the 2D material-based sensors emerges as highly desirable attributes, facilitating their widespread applications in diverse scenarios. Several key challenges such as dynamic strain range control, enhancement of flexibility without compromising other sensing capabilities, adaptability to various body types, long-term durability and reliability, the development of programmable flexibility tailored to specific applications, etc., necessitate further research and development efforts.

### Comprehensive Assessment of 2D Materials for Wearable Device Applications

Given their prolonged attachment to the skin or implanted in the body, wearable devices utilized in health monitoring and human–machine interaction necessitate extensive research into several fundamental concerns regarding biosafety, permeability, and comfort [2]. Unlike many conventional polymer or soft materials with well-determined chemical structures and extensively studied physiochemical properties, emergent 2D materials present new challenges. Specifically, their biosafety remains largely unknown or subject to debate [371], as minute variations in factors such as atomic thickness, surface contamination, functional groups, defective configuration, and lateral size of nanosheets, have been proven to influence their distinctive biosafety characteristics. However, the scattered information is insufficient to provide comprehensive insights for making sound judgments on this open question. Additionally, intact 2D materials are impermeable to molecules [372], which is favorable for device packaging but raises concerns regarding permeability for prolonged skin contact, especially considering additional uncertainties of atomic configuration variations in 2D materials. This necessitates a multidisciplinary approach, combining advancements in materials science, biosafety evaluation, and wearable technology development to enable the safe and comfortable integration of 2D materials in wearable devices.

### Innovative Design Configuration in 2D Material-Based Devices

Building upon the pioneering foundations laid in conventional polymer-based wearable devices, 2D materials are compatible with most of the current device architectures [3], widely serving as conducting electrodes, semiconductive channels, dielectric layers, and more, thereby endowing sensors with compelling performance, harnessing the exceptional properties inherent in 2D nanosheets. However, the incorporation of 2D flakes introduces new concerns, such as dispersion stability/uniformity, rheological variation, interfacial adhesion, processing challenges, mismatch in thermal expansion coefficients, and others [373]. Conversely, the versatile synthetic methods and flexible integration nature in 2D materials facilitate many new device fabrication paradigms like roll-to-roll transfer [374], van der Waals heterostructure [375], all-printing manufacturing [376], laser-induced paternalization [191], etc. These approaches hold great promise for developing new structures and multi-functionalities in advanced wearable sensors in the 2D limit.

### Enhanced Performance in 2D Material-Based Wearable Devices

The competitiveness of 2D materials in wearable device applications hinges on device performance. To underscore the important roles of 2D materials in wearable devices, several key indicators should be comprehensively improved, including security, accuracy, integration, miniaturization, and intelligence [1]. As wearable devices transition from in vitro to in vivo settings [377], elevated standards are imperative to evaluate their operational stability, biological toxicity, and biosafety. Accordingly, research into biosafety packaging materials would be a research focus. In addition, ensuring information security is critical in the era of big data, necessitating encryption measures during data collection and transmission processes. Signal fidelity degradation and noise coupling in signal propagation paths can compromise signal acquisition during monitoring. Address inaccuracies and measurement errors require further efforts, such as encoded transmission [378], machine learning-based filtering algorithms [379], etc., to effectively mitigate confounding signals from factors like sensor displacement or movement of the target tissue. Furthermore, integrated multifunctional sensors demand highly efficient energy management [380] and multi-signal reading/feedback capabilities [381]. Several viable approaches including the incorporation of self-powered modules, wireless power supply modules, and decoupling models in one unit offer viable solutions to this end.

### Versatile Intelligence of 2D Material-Based Wearable Devices

**Fig. 15:**
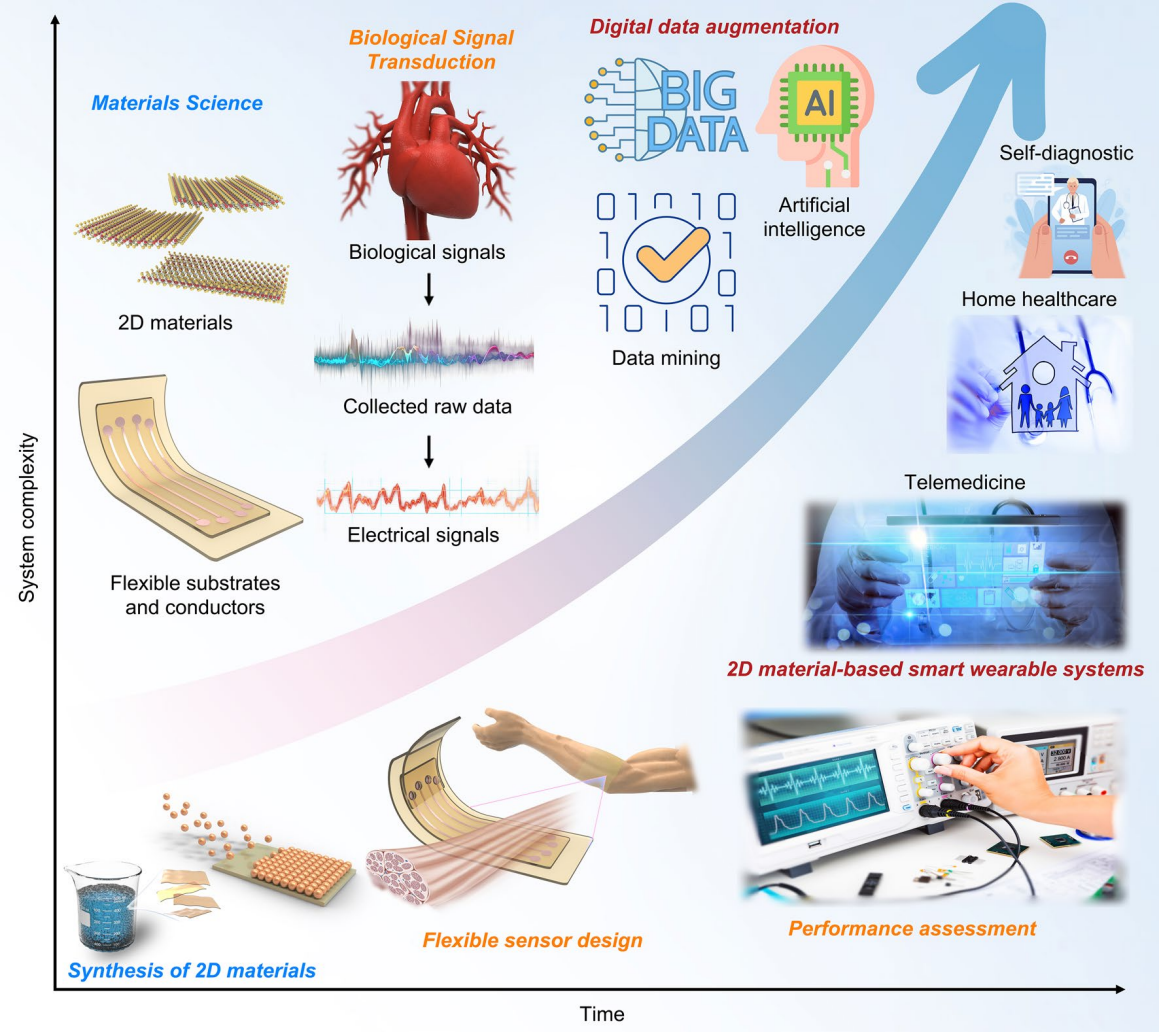
Prospects of 2D material-based wearable biodevices. The next generation of wearable biodevices, from flexible sensors to advanced smart wearable systems, is anticipated to emerge with the advancement of 2D material synthesis methods, flexible sensor design, and performance assessment

Wearable devices based on 2D materials offer promising prospects in fitness monitoring, healthcare, and communication. Currently, flexible wearable sensors are primarily limited to detecting external inputs. However, the human skin’s sensory system not only senses exterior stimuli but also records, processes, and reacts to them. Thus, a significant future research focus lies in enabling flexible wearable sensors to possess skin-like sensing and memory capacities, thereby achieving intelligent perception-a formidable yet crucial endeavor (Fig. 15).

### Market Prospect of 2D Material-Based Wearable Devices

The development of smart sensors presents significant prospects for clinical diagnostics. Fortune Business Insights predictions indicate that the biosensor market will exceed $55.78 billion in 2032 [382]. To fully realize the potential of 2D materials in the field of biosensing, further research and commercialization are necessary. Comprehending the surface chemistry, functionalization techniques, and biocompatibility of these nanosheets is crucial for improving the sensitivity, selectivity, and overall performance of optical biosensors. Additionally, establishing standardized procedures for the reproducible synthesis and functionalization of 2D materials, resolving product inconsistencies, and ensuring the reliability of biosensor results are significant gaps in current research. For widespread commercialization, efforts should focus on standardizing manufacturing processes and enhancing the performance of sensors. Achieving this goal will require collaboration among materials scientists, engineers, and biologists to create commercially viable biosensing systems applicable in fields such as medical diagnostics, drug development, and environmental monitoring. To promote the application of nanomaterials in biodevices, drive innovation, and reduce the research and development cycle from laboratory studies to practical applications, academia, industry, and regulatory bodies must work together to accelerate the filling of market gaps and advance the commercialization of biosensing technologies based on 2D materials.
